# Nucleoside supplements as treatments for mitochondrial DNA depletion syndrome

**DOI:** 10.3389/fcell.2024.1260496

**Published:** 2024-04-02

**Authors:** Eszter Dombi, Tony Marinaki, Paolo Spingardi, Val Millar, Nastasia Hadjichristou, Janet Carver, Iain G. Johnston, Carl Fratter, Joanna Poulton

**Affiliations:** ^1^ Nuffield Department of Women’s and Reproductive Health, University of Oxford, Oxford, United Kingdom; ^2^ Purine Research Laboratory, Department of Biochemical Sciences, Guy’s and St Thomas’ Hospitals, London, United Kingdom; ^3^ Ludwig Institute for Cancer Research, Nuffield Department of Medicine, Medical Sciences Division, University of Oxford, Oxford, United Kingdom; ^4^ Target Discovery Institute, Centre for Medicines Discovery, Nuffield Department of Medicine, University of Oxford, Oxford, United Kingdom; ^5^ Department of Paediatrics, Arch Makarios III Hospital, Nicosia, Cyprus; ^6^ Department of Mathematics, University of Bergen, Bergen, Norway; ^7^ Computational Biology Unit, University of Bergen, Bergen, Norway; ^8^ Oxford Genetics Laboratories, Oxford University Hospitals NHS Foundation Trust, Oxford, United Kingdom

**Keywords:** mitochondrial DNA, mitochondrial DNA depletion syndrome, high-content imaging, nucleoside bypass therapy, heavy isotope labelling mass spectroscopy, alpers syndrome, POLG, TWNK

## Abstract

**Introduction:** In mitochondrial DNA (mtDNA) depletion syndrome (MDS), patients cannot maintain sufficient mtDNA for their energy needs. MDS presentations range from infantile encephalopathy with hepatopathy (Alpers syndrome) to adult chronic progressive external ophthalmoplegia. Most are caused by nucleotide imbalance or by defects in the mtDNA replisome. There is currently no curative treatment available. Nucleoside therapy is a promising experimental treatment for *TK2* deficiency, where patients are supplemented with exogenous deoxypyrimidines. We aimed to explore the benefits of nucleoside supplementation in POLG and TWNK deficient fibroblasts.

**Methods:** We used high-content fluorescence microscopy with software-based image analysis to assay mtDNA content and membrane potential quantitatively, using vital dyes PicoGreen and MitoTracker Red CMXRos respectively. We tested the effect of 15 combinations (A, T, G, C, AT, AC, AG, CT, CG, GT, ATC, ATG, AGC, TGC, ATGC) of deoxynucleoside supplements on mtDNA content of fibroblasts derived from four patients with MDS (POLG1, POLG2, DGUOK, TWNK) in both a replicating (10% dialysed FCS) and quiescent (0.1% dialysed FCS) state. We used qPCR to measure mtDNA content of supplemented and non-supplemented fibroblasts following mtDNA depletion using 20 µM ddC and after 14- and 21-day recovery in a quiescent state.

**Results:** Nucleoside treatments at 200 µM that significantly increased mtDNA content also significantly reduced the number of cells remaining in culture after 7 days of treatment, as well as mitochondrial membrane potential. These toxic effects were abolished by reducing the concentration of nucleosides to 50 µM. In POLG1 and TWNK cells the combination of ATGC treatment increased mtDNA content the most after 7 days in non-replicating cells. ATGC nucleoside combination significantly increased the rate of mtDNA recovery in quiescent POLG1 cells following mtDNA depletion by ddC.

**Conclusion:** High-content imaging enabled us to link mtDNA copy number with key read-outs linked to patient wellbeing. Elevated G increased mtDNA copy number but severely impaired fibroblast growth, potentially by inhibiting purine synthesis and/or causing replication stress. Combinations of nucleosides ATGC, T, or TC, benefited growth of cells harbouring *POLG* mutations. These combinations, one of which reflects a commercially available preparation, could be explored further for treatment of POLG patients.

## Introduction

Mitochondrial DNA depletion syndrome (MDS) is an umbrella term for a constantly expanding group of human disorders that result from defects in mitochondrial DNA (mtDNA) maintenance. The mitochondrial genome encodes 13 proteins, all of which are key catalytic subunits of the electron transport chain. Therefore, mtDNA depletion is detrimental to oxidative phosphorylation and hence directly impacts patient health. These diseases are characterised by reduced mtDNA copy number in affected tissues and cause clinical syndromes ranging from pure myopathy to encephalopathy with hepatopathy (Alpers syndrome). Mutations in numerous nuclear genes impair mtDNA maintenance, most commonly *POLG, TWNK, DGUOK*, and *TK2*. *POLG* and *TWNK* are directly involved in mtDNA replication (Rotig and Poulton, 2009; Suomalainen and Isohanni, 2010). Polymerase gamma (poly γ) is the key enzyme of the mitochondrial replisome, comprising a catalytic subunit with exonuclease activity encoded by the *POLG* gene, and an accessory subunit encoded by *POLG2* which enhances binding to DNA as well as processivity (Copeland, 2008). Poly γ cooperates with the mitochondrial 5′-3′ helicase TWINKLE, encoded by the *TWNK* gene, necessary for mtDNA replication (Wanrooij et al., 2007). DGUOK and TK2 are mitochondrial kinases that mediate the first step of the deoxy-purine and deoxy-pyrimidine salvage pathway respectively, hence affecting mtDNA replication indirectly via dNTP availability. Defects of the replisome (Ashley et al., 2008) and dNTP imbalance (Ashley et al., 2007) both cause replication stalling which slows synthesis and introduces point mutations into mtDNA. This is a type of “mtDNA replication stress”, defined as conditions that interfere with DNA replication and hamper its progression. Furthermore, cells harbouring a severe *POLG* exonuclease domain mutation may exert replication stress on the nucleus by sequestering nucleotides (Hamalainen et al., 2019) because of increased mtDNA synthesis and turnover (Lodge et al., 2017).

Efficient mitochondrial DNA replication and maintenance requires a balanced supply of deoxynucleoside triphosphates (dNTPs), which are provided by a wide range of anabolic and catabolic enzymes. Deoxynucleotides are either synthesized *de novo* in the cytosol or acquired via the salvage pathway. Enzymes for salvage are located within mitochondria and in the cytosol (Lane and Fan, 2015). *De novo* nucleotide synthesis starts from a variety of precursors resulting in ribonucleoside diphosphates that are reduced to their corresponding deoxyribonucleoside diphosphates by ribonucleotide reductases (RNRs) (Nordlund and Reichard, 2006). The *de novo* synthesis pathway is mainly active during S-phase and provides dNTPs for both nuclear and mitochondrial genome replication in proliferating cells (Nordlund and Reichard, 2006). RNRs are regulated by the cell cycle and are present at very low amounts in non-replicating cells (Hakansson et al., 2006) Therefore, terminally differentiated or quiescent cells likely depend on the salvage pathway for a balanced supply of dNTPs for mtDNA replication/repair, producing these dNTPs from recycled deoxynucleosides. The recycled deoxynucleosides are phosphorylated by thymidine kinase 1 (TK1) and deoxycytidine kinase (dCK) in the cytosol and thymidine kinase 2 (TK2) and deoxyguanosine kinase (dGK) in the mitochondria. Mutations in both mitochondrial kinases (*TK2* and *DGUOK*) have been linked to MDS (Mandel et al., 2001; Saada et al., 2001).

Ever since the first experiments restoring mtDNA copy number with nucleotide supplements in *DGUOK* mutant cells that were quiescent and hence dependent on their defective dGK (Taanman et al., 2003), supplementation has been explored as a treatment (see Table 1). In the case of TK2 deficiency, supplementation with dCMP + dTMP has been successful *in vivo* (Garone et al., 2014; Amtmann et al., 2023). But the response to supplementation is determined by the precise molecular lesion. For instance, positive responses to supplementation have been documented in POLG deficient myoblasts (Bulst et al., 2012) and fibroblasts (Blazquez-Bermejo et al., 2019) (Table 1) but not yet in TWNK deficient cells. While previous studies focused on a single combination of deoxynucleotides, we sought to explore a wide range of deoxynucleoside combinations in cellular models of mtDNA depletion syndrome due to *POLG* and *TWNK* deficiency. We used high-content imaging and a combination of three readouts; mtDNA content, cell number and mitochondrial membrane potential to select the most promising deoxynucleoside combination to investigate further. Following the lead of previous studies, we used dialysed foetal calf serum (FCS) to reduce exogenous nucleotide levels in the culture medium and investigated both cycling cells cultured in 10% foetal calf serum as well as quiescent cells cultured in 0.1% FCS to model post-mitotic tissues. We also included *DGUOK* mutant fibroblasts because these manifest a well-documented increase in mtDNA content in response to supplementation (Table 1 (Taanman et al., 2003; Saada, 2008; Bulst et al., 2009)).

**TABLE 1:** Summary of deoxynucleoside/nucleotide supplementation publications using nucleoside bypass therapy in *in vitro* models of MDS.

References	*Gene defecs investigated*	Model	Treatment regime	Serum starvation	Outcome measure
**Taanman et al., 2003, Human Molecular Genetics** (Taanman et al., 2003)	*DGUOK* (homozygous nonsense mutation in exon 3)	patient derived fibroblasts	deoxyguanosine monophosphate (dGMP) and deoxyadenosine monophosphate (dAMP) @200 uM (uridine still in medium potentially, complete serum starvation)	0–40 days serum withdrawal during treatment	mtDNA conent by Southern blot
					cytochrome-c oxidase subunit II expression level
**Saada et al., 2008, Molecular Genetics and Metabolism** (Saada, 2008)	*DGUOK* (homozygous 204delA deletion)	patient derived fibroblasts	10 days treatment with a combination of deoxy guanosine (dGuo) and deoxyadenosine (dAdo) @ 100 uM (uridine still in medium potentially, complete serum starvation)	10 days complete serum starvation (pre-treatment)	mtDNA content by qPCR
					enzymatic activities of MRC complexes
					dCK activity
**Bulst et al., 2009, Human Molecular Genetics** (Bulst et al., 2009)	*DGUOK* (homozygous c.705þ1_4delGTAA), *DGUOK* (homozygous p.52S.F)	patient derived myoblasts and myotubes	dGMP and dAMP @50, 100, 200, 400, 800, 1,200 uM or uridine only for 6 days in reduced serum fusion medium (2% horse serum). Note: toxicity reported @1200 uM manifesting in decreased fusion capacity	6 days 2% horse serum during treatment	mtDNA content by qPCR
	*POLG1* (compound heterozygous p.Ala467Thr/p.Lys1191Asn)				
	*TYMP* (homozygous p.Glu289Al)				
**Bulst et al., 2012, Molecular Genetics and Metabolism** (Bulst et al., 2012)	*POLG*	patient derived myoblasts/MyoD transfected patient fibroblasts	dAMP/dGMP or with a mixture of all dNMPs (dAMP/dCMP/dGMP/dTMP) @200 and 400uM	6 days 2% horse serum during treatment	mtDNA content by qPCR
	*RRM2B*				
**Gonzalez-Vioque et al., 2011, PLOS Genetics** (Gonzalez-Vioque et al., 2011)	*TYMP*	*In organello* (mouse liver) model	dATP, dTTP, dGTP, dCTP @1uM and 100uM	2 h	Endogenous intramitochondrial deoxynucleotide content
					Radiolabelled deoxynucleotide incorporation to mtDNA
**Bl´azquez-Bermejo et al., 2019, The FASEB Journal** (Blazquez-Bermejo et al., 2019)	*POLG*	patient derived fibroblasts	combination of all four nucleosides @50uM ( ± EHNA) following 10 days forced depletion with EtBr	0.1% dialyzed FCS pre-treatment for 3 days	mtDNA content by qPCR
**Current study**	*POLG*	*Patient derived fibroblasts*	Deoxyadenosine, deoxythimidine, deoxyguanosine, deoxycytidine alone or in all possible combinations (A, T, G, C, AT, GC, AG, TC, AC, GT, ATG, ATC, AGC, TGC, ATGC	3 days pre-treatment 0.1% than 7 days supplement	mtDNA content (PicoGreen), membrane potential, cell count, deoxynucleoside incorporation
	*TWNK*				
	*DGUOK*				

The italics denote the genotype

## Materials and methods

### Cell culture

All materials for the maintenance of fibroblasts were purchased from Merck (formerly Sigma-Aldrich) unless otherwise stated.

Patient skin biopsies were performed following informed consent, with the approval of the United Kingdom National Research Ethics Service (South Central-Berkshire). Human fibroblast cultures were established from skin explants following standard procedure. Cells were maintained in high-glucose Dulbecco’s modified Eagle’s medium (DMEM) supplemented with 10% FCS, 100U/mL penicillin and 100 μg/mL streptomycin, 2 mM L-glutamine and 50 μg/mL uridine at 37°C in a humidified incubator with 5% CO_2_. Media was changed every 3–4 days. Cells were routinely cultured in T25/T75 tissue culture flasks and split when reached ∼80–90% confluency. Prior to the experiments, cells were tested for possible *Mycoplasma* contamination using the EZ-PCRTM *Mycoplasma* Test Kit (Geneflow) following the manufacturer’s instructions.

### Galactose viability

Fibroblasts were seeded into six well plates at a density of 20,000 cells per well. Cells were left to attach overnight. The next day (day 0) the media was changed to either high-glucose maintenance media or glucose free DMEM (Thermo Fisher Scientific, Gibco) supplemented with 10% FCS, 100U/mL penicillin and 100 μg/mL streptomycin, 1 mM sodium pyruvate, 50 μg/mL uridine and 10 mM galactose. Media was changed on day 3. On day 7 the cells were trypsinized and counted manually using a haemocytometer. The number of cells remaining in galactose media was expressed as the percentage of the cells counted in high-glucose media (Mortiboys, 2006).

### High-content fluorescent microscopy

#### PicoGreen and TMRM staining

Fibroblasts were plated into black optical bottom 96-well tissue culture treated plates (PerkinElmer) at a density of 3 × 10^3^ cells/well. The cells were left to attach overnight, and media was changed the next day (100 µL/well). 24 h later fibroblasts were stained following a protocol adopted from Ashley et al., 2005 (Ashley et al., 2005; Uusimaa et al., 2014), who validated it for the detection of mtDNA content within live cells. Quant-iT™ PicoGreen™ dsDNA Reagent (Thermo Fisher Scientific) was received as a stock solution in DMSO. The cells were simultaneously co-stained with the mitochondrial membrane potential sensitive vital dye, Tetramethylrhodamine, Methyl Ester, Perchlorate (TMRM, Merck). TMRM was received as a powder and was diluted to a 50 μM stock in DMSO. For live cell staining, PicoGreen and TMRM stocks were diluted in cell culture media 1:333 (3 μL/mL) and 1:2000 respectively. 100 μL of staining solution was added to each well of a 96-well plate. Fibroblasts were incubated in the staining solution for 45 min at 37°C in a humidified incubator with 5% CO_2_. The staining solution was removed and replaced with 150 μL pre-warmed reduced serum Opti-MEM (Thermo Fisher Scientific) for imaging.

#### PicoGreen and MitoTracker red CMXRos staining

MitoTracker Red CMXRos (Thermo Fisher Scientific) was received as a powder and was diluted to a 1 mM stock in DMSO and stored at −20°C in aliquots. For live cell staining, PicoGreen and CMXRos stocks were diluted in cell culture media 1:333 (3 μL/mL) and 1:10000 respectively. 100μL of staining solution was added to each well of a 96-well plate. Fibroblasts were incubated in the staining solution for 30 min at 37°C in a humidified incubator with 5% CO2. The staining solution was removed and replaced with 150 μL pre-warmed reduced serum Opti-MEM (Thermo Fisher Scientific) for imaging.

#### Image acquisition

Plates were imaged using an INCell 1000 automated microscope (GE Healthcare). Images were taken in nine randomly distributed fields in each well of a 96-well plate. The position of each field remained constant in between wells. Image stacks were analysed by an in-house written protocol using the INCell Developer software (GE Healthcare) (Uusimaa et al., 2014; Diot et al., 2015).

#### Image analysis

One key advantage of high-content imaging is that the PicoGreen signal from both nucleus and nucleoids can be measured in the same cell, by segmenting on size (Uusimaa et al., 2014). Measurements of differences within a run are more robust than of differences between runs. To investigate mtDNA content we focussed on both PicoGreen puncta numbers and summed area of puncta per cell, as previously (Ashley et al., 2005). We assessed mitochondrial membrane potential using the integrated density of the TMRM signal (the product of intensity and mitochondrial area, TMRM signal henceforth referred to as “mitochondrial membrane potential”). Using this quantity, we assessed differences in the mass of polarised mitochondria between cells by making comparisons of different treatments largely within a particular primary culture. We also measured nuclear mass as the integrated density (product of nuclear area and intensity) of PicoGreen signal. Observation of the cell-to-cell distribution of these quantities revealed that they typically follow a normal distribution, with a variance that itself varies between individual cultures.

### Supplementation with deoxyribonucleosides

For experimental purposes regular foetal calf serum was replaced with dialysed foetal calf serum throughout the experimental procedure (dFCS, Thermo Fisher Scientific). Cells were treated in high-glucose DMEM media (Merck) supplemented with 100U/mL penicillin and 100 μg/mL streptomycin (Merck), 2 mM L-glutamine (Merck) and either 10% or 0.1% dialysed foetal calf serum (Thermo Fisher Scientific), no uridine. Control and patient derived fibroblasts were seeded into 96-well optical clear bottom tissue culture treated plates (Perkin Elmer) at a density of 5 × 10^2^ cells/well for culture with 10% dFCS and 3 × 10^3^ cells/well for reduced serum 0.1% dFCS culture. Fibroblasts were supplemented with all possible combinations of deoxynucleosides (A, T, G, C, AT, AC, AG, CT, CG, GT, ATC, ATG, AGC, TGC, ATGC, Sigma-Aldrich), each at either 50 or 200 µM concentration for 7 days. Deoxynucleosides were replenished on day 3. Fibroblasts cultured in 0.1% dFCS were pre-conditioned in low serum medium for 3 days (d-3) to stop nuclear replication and therefore mimic affected post-mitotic tissue. On day 7 the plates were stained and imaged as described above.


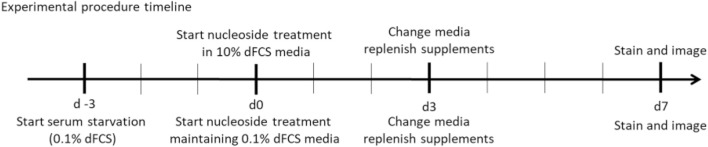



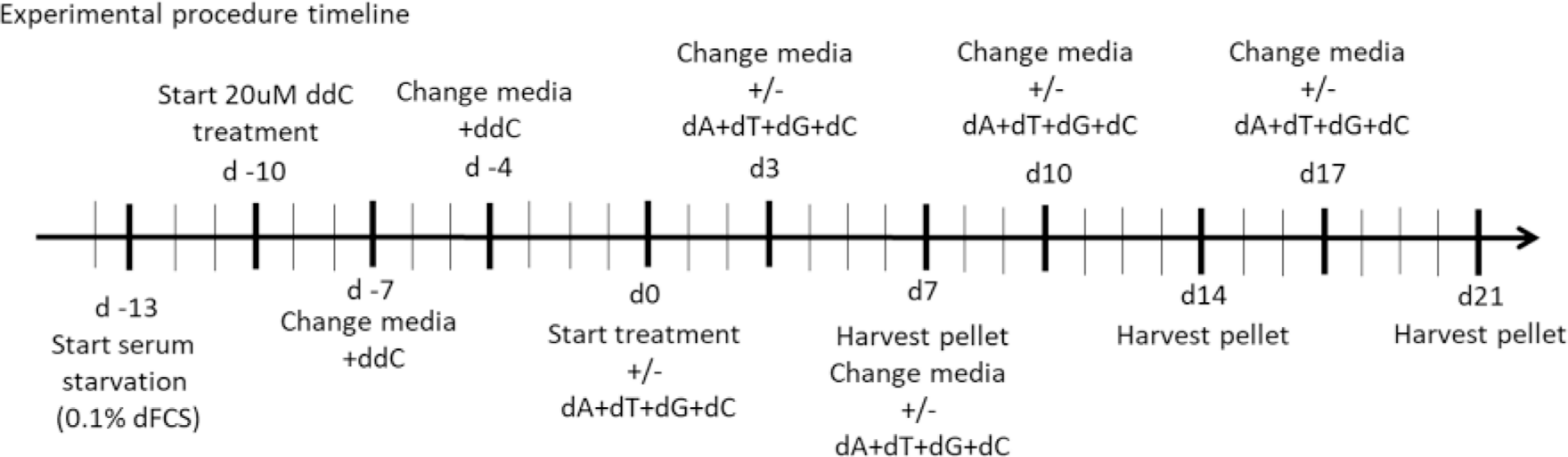


### Mitochondrial DNA depletion and recovery with deoxyribonucleosides

As previously, for experimental purposes regular foetal calf serum was replaced with dialysed serum throughout the experimental procedure (dFCS, Thermo Fisher Scientific). Cells were treated in high-glucose DMEM media (Merck) supplemented 100U/mL penicillin and 100 μg/mL streptomycin (Merck), 2 mM L-glutamine (Merck) and 0.1% dialysed foetal calf serum (Thermo Fisher Scientific), no uridine.

Control and *POLG*, *TWNK* and *DGUOK* deficient fibroblasts were seeded into 8×T25 (per cell culture) tissue culture treated flasks in high-glucose DMEM media containing 10% dFCS at a density of 2 × 10^6^ cells/flask (d −14). The next day, the media was replaced by high-glucose DMEM containing 0.1% dFCS (d −13). High-glucose DMEM with 0.1% dialysed serum was used for the remainder of the experimental procedure. 72 h later (d −10) 7xT25 flasks/cell culture were treated with 20 µM 2′-3′-dideoxycytidine (ddC, Sigma-Aldrich) for 10 days. Media with ddC was changed on d −7 and d −4. 1×T25 flask/cell culture was kept as untreated control. After 10 days the cells from the untreated flasks and 1× ddC treated flask were harvested as reference pellets (d0). The remaining six flasks/cell culture were split into 3× untreated (DMSO control) and 3× treated. The treated flasks were supplemented with the combination of all four deoxynucleosides (ATGC) each at a concentration of 50 µM. Media was changed every 3–4 days. A pair of treated and untreated flasks were harvested after 7 days (d7), 14 days (d14) and 21 days (d21) of treatment.

The experimental procedure was repeated with each cell culture on 4 separate occasions. On two occasions the cells were supplemented with regular deoxyribonucleosides. These samples were used for subsequent quantification of mtDNA content. On two occasions the deoxynucleoside supplement mix consisted of 90% regular and 10% heavy labelled deoxynucleosides 13C A, 13C C, 15N13C T and 15N G were purchased from Silantes. These samples were used to quantify the incorporated deoxyribonucleosides by mass spectroscopy.

### DNA isolation

Total cellular DNA was isolated using the GenElute Mammalian Genomic DNA Miniprep Kit (Sigma-Aldrich) following manufacturer’s instructions.

### Quantification of heavy isotope labelling by Mass Spectroscopy

Hydrolysis was carried out in 50 µL reactions that contained 50 ng DNA, 100 mM NaCl, 20 mM MgCl_2_, 20 mM Tris pH 7.9, 1000 U/mL Benzonase, 600 mU/mL Phosphodiesterase I, 80 U/mL Alkaline phosphatase, 36 μg/mL EHNA hydrochloride and 2.7 mM deferoxamine for 2 h at 37°C. Following lyophilisation, nucleosides were resuspended in 50 µL buffer A (10 mM ammonium acetate, pH 6).

For the analysis by HPLC–QQQ mass spectrometry, a 1,290 Infinity UHPLC was fitted with a Zorbax Eclipse plus C18 column, (1.8 µm, 2.1 mm 150 mm; Agilent) and coupled to a 6495a Triple Quadrupole mass spectrometer (Agilent Technologies) equipped with a Jetstream ESI-AJS source. The data were acquired in dMRM mode using positive electrospray ionisation (ESI1). 5 μL of the samples were injected per run. The gradient used to elute the nucleosides started by a 5 min isocratic gradient composed of 100% buffer A and 0% buffer B (100% methanol) with a flow rate of 0.4 mL/min and was followed by the subsequent steps: 5–8 min, 94.4% A; 8–9 min, 94.4% A; 9–16 min 86.3% A; 16–17 min 0% A; 17–21 min 0% A; 21–24.3 min 100% A; 24.3–25 min 100% A. The AJS ESI settings were as follows: drying gas temperature 230°C, the drying gas flow 14 lmin-1, nebulizer 20 psi, sheath gas temperature 400°C, sheath gas flow 11 lmin-1, Vcap 2,000 V and nozzle voltage 0 V. The iFunnel parameters were as follows: high pressure RF 110 V, low pressure RF 80 V. The fragmentor of the QQQ mass spectrometer was set to 380 V and the delta EMV set to +200.

The raw mass spectrometry data was analysed using the MassHunter Quant Software package (Agilent Technologies, version B.08.01). The transitions and retention times used for the characterization of nucleosides and their adducts are summarised in Supplementary Table S1. For the identification of compounds, raw mass spectrometry data was processed using the dMRM extraction function in the MassHunter software.

### MtDNA copy number analysis

In addition to using high throughput imaging to estimate mitochondrial DNA copy number, we confirmed key findings by using real-time quantitative PCR, using primers and probes specific for mitochondrial DNA and the single copy nuclear gene, *B2M*, assayed simultaneously using a PE7500 real-time PCR instrument (Applied Biosystems, Thermo Fisher Scientific, Waltham, MA, United States) (primer and probe sequences available online at https://github.com/StochasticBiology/mtdna-nucleosides).

### Statistical analysis

Normality for the distribution of each sample in each condition was assessed using a Shapiro-Wilk test. Differences from baseline were assessed with Mann-Whitney *U* test where the distribution differed from normality and t-tests were used where there was no evidence against normality, IBM SPSS statistics version 28 was used to carry out the analysis. Error bars are standard errors unless otherwise stated to account for differences between runs and cell lines when amalgamating data, linear mixed models (LMMs) were used for some analyses, assigning random effects to run (and cell line where appropriate). These additional statistical analysis were performed in R (R C Team, 2022) using libraries ggplot2 (Wickham, 2016), ggrepel (S K, 2021), readxl (Wickham H, 2023) nlme (Pinheiro et al., 2022) and lme4 (Bates et al., 2015). Code and data for this analysis is freely available online at https://github.com/StochasticBiology/mtdna-nucleosides.

## Results

### Mitochondrial DNA content of fibroblasts derived from patients with MDS due to different genetic defects

**FIGURE 1:**
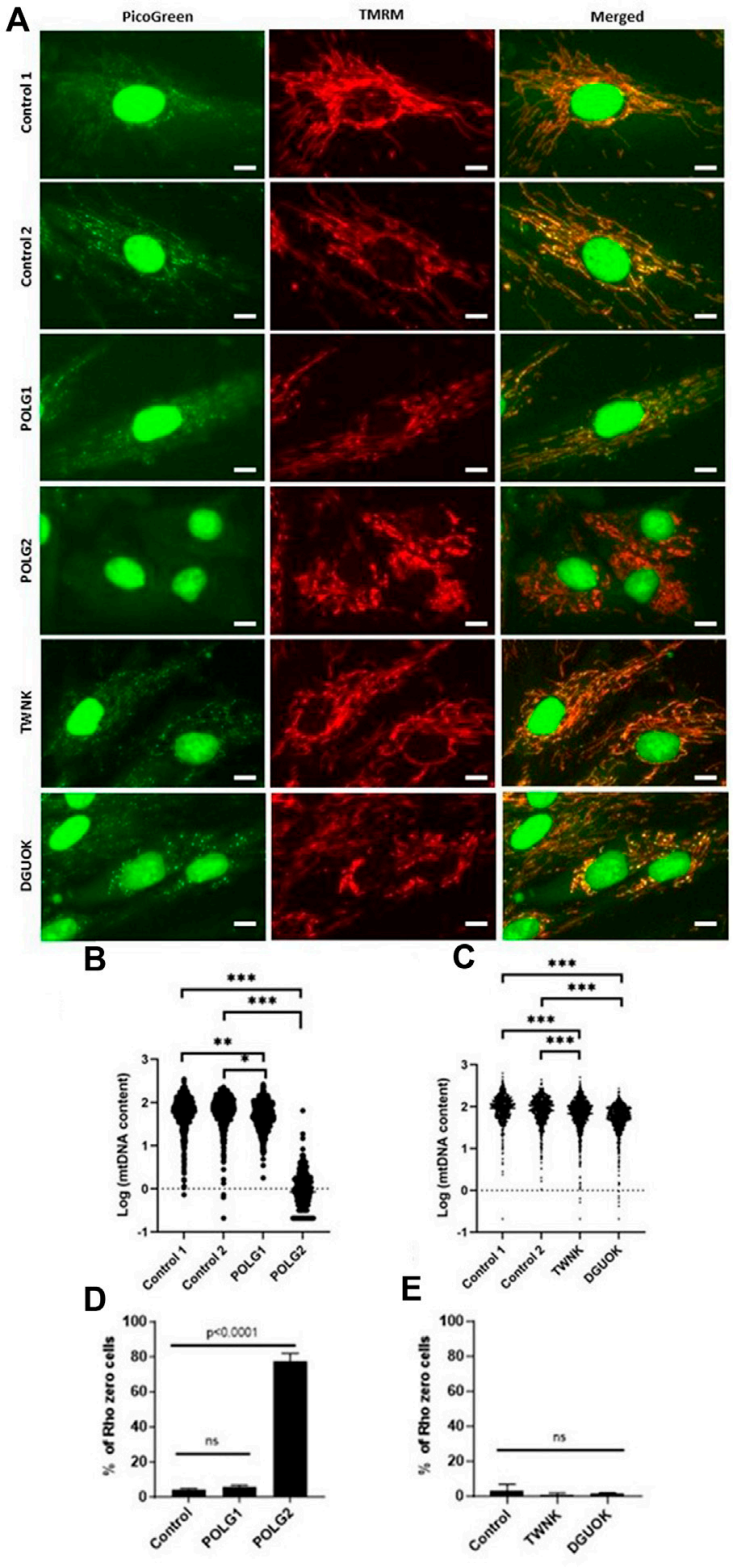
MtDNA content of control and patient derived fibroblasts measured by high-content imaging. **(A)** Representative images of Control 1, Control 2, POLG1, POLG2, TWNK and DGUOK fibroblasts co-labelled with PicoGreen and TMRM (see also Supplementary Figure S1B). Scale bar 10 uM **(B, C)** Quantification of mtDNA content in patient and control cells in baseline growth conditions expressed as Log (Nordlund and Reichard, 2006) plot of the sum area of mtDNA nucleoids labelled by PicoGreen,. **(D, E)** Percentage of Rho 0 cells detected in each culture. Normality was tested with Shapiro-Wilk *p < 0.05, **p < 0.01, ***p < 0.001 (Mann-Whitney *U* test when normality was rejected, t-test otherwise).

Patient and control fibroblast cultures used in this study are summarised, with a short clinical description in Table 2. We selected three controls from our bank of 14 anonymised control fibroblast cultures comprising five children with normal cytogenetics undergoing diagnostic skin biopsy for karyotyping and nine healthy consented adults aged 18–81 years. Control 1 was selected being from the paediatric age range and Controls 2 and 3 are adults who are near to the median for most of the readouts we measure. We studied two patients who were both compound heterozygotes for mutations in *POLG*, encoding the catalytic subunit of the mitochondrial DNA polymerase gamma. Patient POLG1 presented at age 2, mitochondrial DNA depletion in liver (26% mtDNA content) and reduced mtDNA levels in muscle (64% mtDNA content). Patient POLG2 presented as an infant and profound mtDNA depletion was reported in both liver (9% mtDNA content) and muscle (9% mtDNA content). We investigated the mitochondrial DNA content of patient fibroblasts using high-content imaging of live cells labelled with the fluorescent nucleic acid stain PicoGreen (Ashley et al., 2005) (Figure 1A), a method we have validated against qPCR (Uusimaa et al., 2014). We compared the mtDNA content of patient cells with fibroblasts derived from a paediatric (Control 1) and an adult (Control 2) control. The mtDNA content of POLG1 cells was reduced by ∼20% when compared to the control cells, while POLG2 cells represent an extreme case and are almost completely devoid of mtDNA and of mitochondrially encoded proteins (Figure 1B; Supplementary Figures S1A–C). Two additional fibroblast lines included were derived from a patient with a homozygous mutation in the mitochondrial helicase Twinkle (*TWNK*) and one with defects in deoxyguanosine kinase (*DGUOK*), an essential component of the mitochondrial nucleotide salvage pathway. We included patient TWNK in this study because to our knowledge the response of TWNK deficient cells have never been investigated in deoxynucleoside supplementation studies. Additionally, we choose patient DGUOK because these cells are expected to respond to supplementation (see Table 1 for references). When measured with PicoGreen, TWNK and DGUOK fibroblasts had a lower mtDNA content compared to Control 1 cells, which was a statistically significant (all *p* < 0.001, Mann-Whitney *U* test) but small magnitude effect (Figure 1C). We measured mosaic mtDNA depletion by quantifying the proportion of Rho 0 cells, those void of mtDNA, in each culture. Mosaic mtDNA depletion was mild in POLG1 cells and profound in POLG2 cells (Figure 1D); the proportion of profoundly mtDNA depleted cells was sometimes as high as 80% in culture POLG2 (Figure 1D). Not surprisingly, when investigated by Western blot analysis respiratory chain components were significantly reduced in POLG2 cells (Supplementary Figure S1A). TWNK and DGUOK cells had no detectable increase in Rho 0s when compared to the controls (Figure 1E).

**TABLE 2:** Fibroblast cultures in this study.

Patient Identifier​	Mutation	Age	Short clinical description
POLG1	*POLG*	Paediatric​	2-year-old male presented with developmental delay, epilepsy and acute liver failure while on valproate. MtDNA copy number was 26% in liver and 64% in muscle.
	Compound heterozygous c.2243G>C p. (Trp748Ser) & c.2125C>T p. (Arg709*)		
POLG2	*POLG*	Paediatric​	Infant male presented with hypotonia, hypoglycaemia, micrognathia, failure to thrive, raised blood lactate (4.9) and steatotic liver. Muscle respiratory chain enzyme analysis showed reduced activities of complex I and IV. MtDNA copy number was 9% in muscle and 9% in liver, Profound mtDNA depletion in fibroblasts.
	Compound heterozygous c. [752C>T; 1760C>T] p. [(Thr251Ile; Pro587Leu)] & c.3406G>A p. (Glu1136Lys)		
TWNK	*TWNK*	Paediatric (died 10w)	Infant male died at 10 weeks of age. Muscle biopsy showed accumulation of lipid droplets in both Type 1 and Type 2 fibres with a small fibre diameter. MtDNA copy number was 27% in muscle.
	Homozygous c.1183T>C p. (Phe396Leu)		
DGUOK​	*DGUOK*	Paediatric​	Infant female presented with liver failure, renal failure, lactic acidaemia, and hypoglycaemia. Brain MRI showed basal ganglia changes. Muscle mtDNA copy number was 43% with normal respiratory chain enzyme analysis.
	Compound heterozygous c.591G>A (pathogenic splice) & c.757_759delAAT p. (Asn253del)		
Control 1	healthy control	Paediatric​	Male Aged 6y
Control 2	healthy control	Adult	Male Aged 21y
Control 3	healthy control	Adult	Male Aged 21y

### Mitochondrial membrane potential and cell growth in galactose medium as measures of mitochondrial function

**FIGURE 2:**
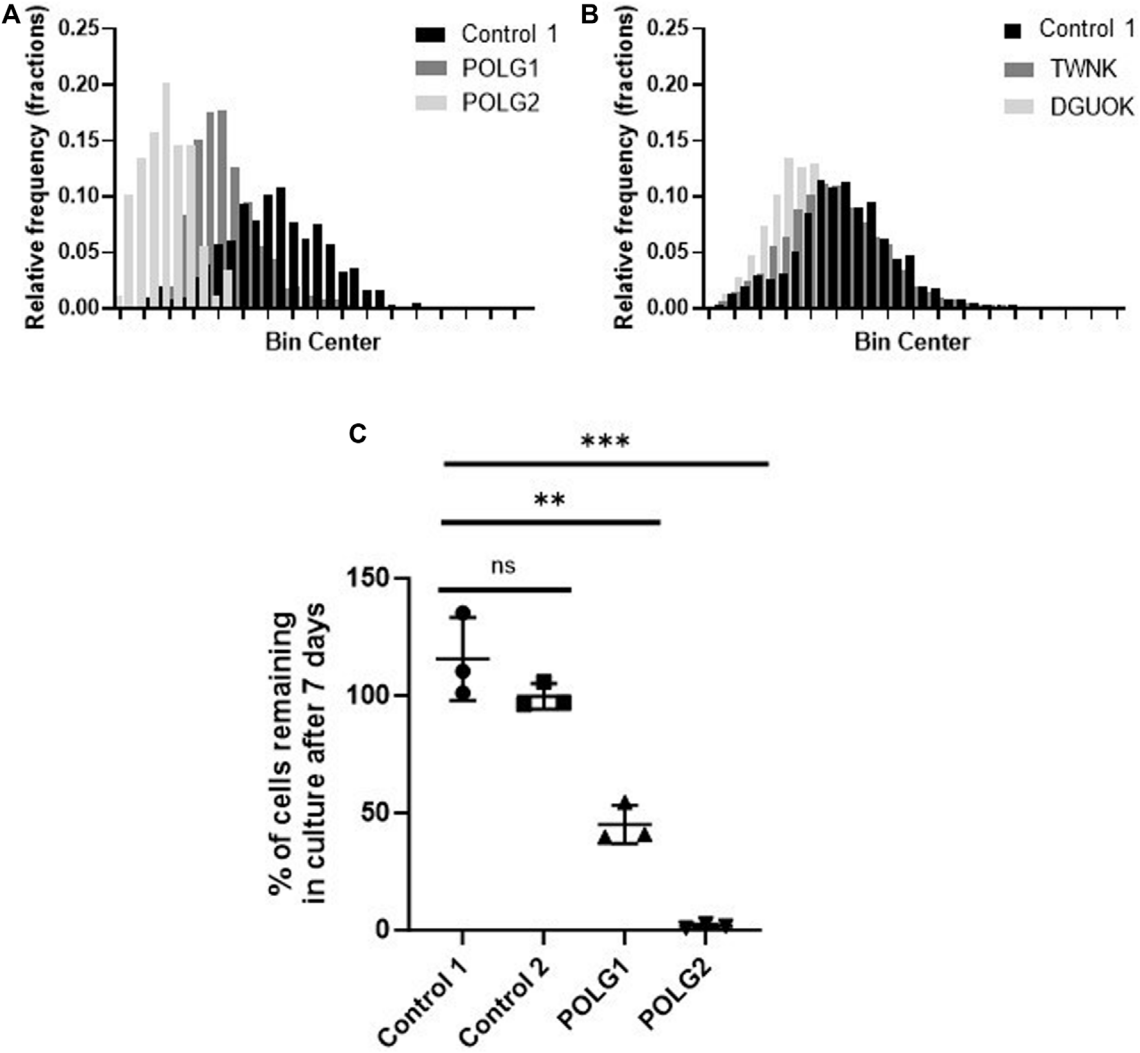
Mitochondrial membrane potential and glucose dependence of control and patient fibroblasts. **(A, B)** Frequency distribution of mitochondrial membrane potential of individual cells measured by staining the cells with the live cell dye TMRM and subsequent high-content imaging, consistent with Figure 1A and Supplementary Figure S1B. POLG2 cells have a significantly reduced signal due to depolarisation compared to Control 2 and to fibroblasts from patient POLG1 (*p* < 0.01). *n* = 3 technical replicates. **(C)** Growth of POLG mutant cells in galactose medium. Number of cells remaining in culture after 7 days expressed as the percentage of cells cultured in high-glucose medium. N = 3 biological replicates, mean with SD, (unpaired t-test, ** *p* < 0.01, *** *p* < 0.001)

Mitochondrial membrane potential of control and patient cells was measured alongside mtDNA content by high-content imaging using the vital dye TMRM that accumulates in the matrix compartment of the organelle. The histogram of TMRM signal measured on a cell-by-cell basis suggested an increase in depolarised mitochondria in both POLG1 and POLG2 cells when compared to Control 2 (Figure 2A), entirely consistent with the appearance by eye (Figure 1A; Supplementary Figure S1B). The TMRM signal in TWNK and DGUOK fibroblasts were similar to Control 2 cells (Figure 2B), with the DGUOK cells being slightly more depolarised. Comparisons of cell growth under energetic stress is a useful surrogate for mitochondrial function in severely compromised cells (Mortiboys, 2006). To investigate the respiratory function of the depolarised POLG1 and POLG2 cultures, we cultured these cells under energetic stress in glucose-free DMEM supplemented with 10 mM galactose. The cells were counted after 7 days and counts in the galactose culture were expressed as the proportion of cells cultured in high-glucose medium (Figure 2C). Because of their profound mtDNA depletion POLG2 cells are highly dependent on glucose, and most cells died in galactose, while the number of cells remaining in culture was reduced to ∼50% in POLG1 (Figure 2C).

### Deoxynucleoside supplementation at high concentration results in substantial toxicity in cycling POLG, TWNK and control cells

**FIGURE 3:**
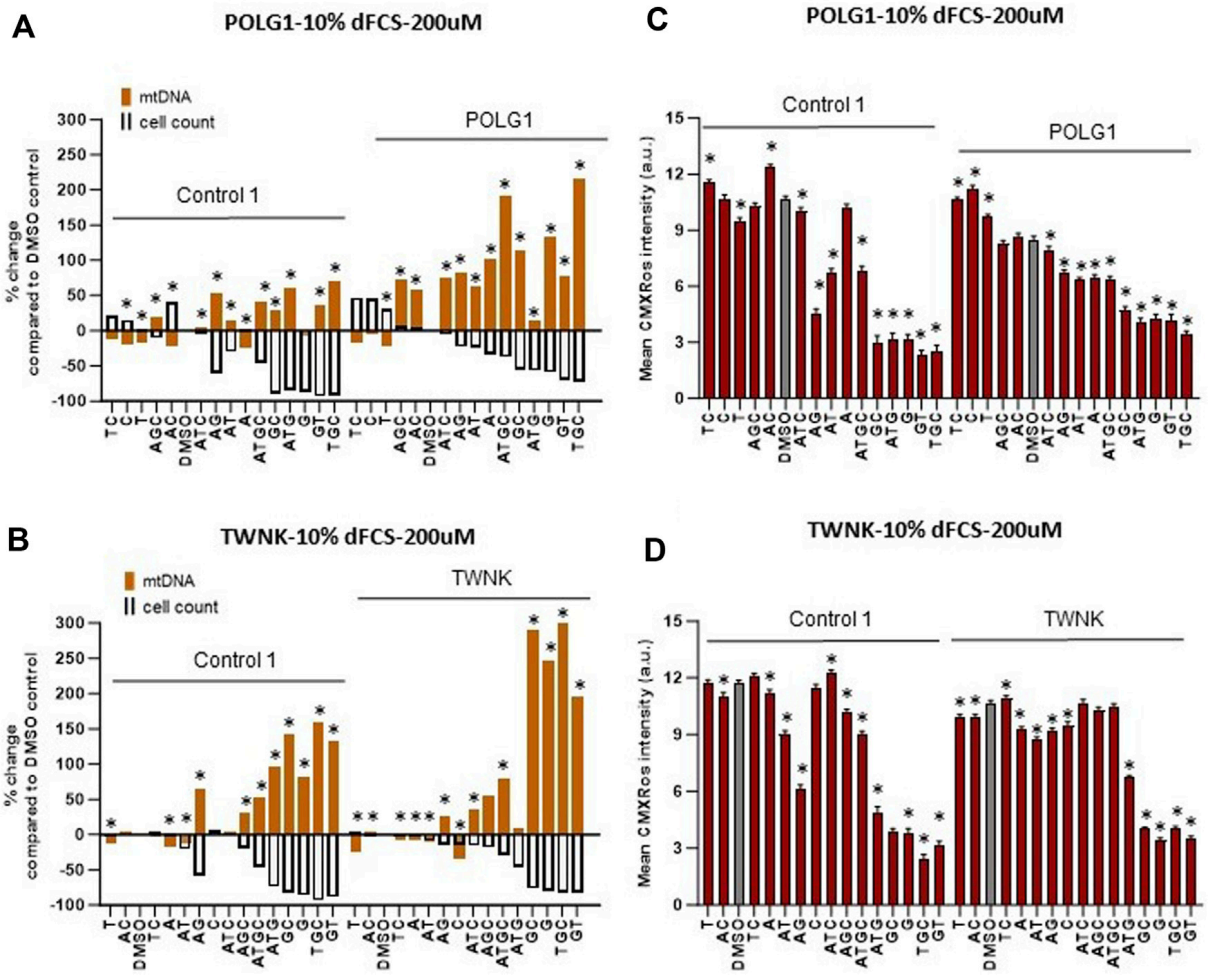
Deoxynucleoside supplementation of cycling cells (10% dFCS) at high concentration (200 µM). **(A, B)** Effect of different nucleoside combinations on mtDNA content and cell count in **(A)** POLG1 and **(B)** TWNK. Effects are presented as average percentage change relative to DMSO baseline. Error bars are omitted because this ratiometric presentation of effects leads to large uncertainties. *, *p* < 0.05 compared to DMSO baseline using Mann-Whitney test. Supplementary Figure S3 shows patterns of statistical significance and number of cells in each sample. Further analysis with linear mixed models is used to assess statistical significance of group effects (see text). **(C, D)** CMXRos intensity for corresponding conditions in both patient **(C)** POLG1 and **(D)** TWNK. Significant values increase in mtDNA over baseline for illustrated run are indicated by * *p* < 0.05, (Mann-Whitney *U* test). All averages are amalgamated in scatterplots in Supplementary Figure S5.

We supplemented our patient cells with exogenous deoxynucleosides and subsequently measured mtDNA content and membrane potential using high-content imaging as previously (Diot et al., 2015) to establish any potential benefit to the cells. For the remainder of this study, we used the vital dye MitoTracker Red CMXRos, rather than TMRM to measure mitochondrial membrane potential. We previously validated PG/TMRM (Uusimaa et al., 2014), but not PG/MitoTracker Red CMXRos (henceforth “CMXRos”) staining as a readout for mtDNA content. We therefore compared CMXRos with TMRM. Like TMRM, CMXRos is a red-fluorescent dye that stains mitochondria in live cells and its accumulation is dependent upon membrane potential. It has the advantage of being suitable for fixation and manifests less run-to-run variability than TMRM. Measurements of mtDNA copy number (summed area of PicoGreen mtDNA signal) under different treatments within a culture counterstained with TMRM correlated well with PicoGreen signal counterstained with CMXRos (Supplementary Figure S2). Previous studies (Table 1) used deoxynucleoside monophosphate supplements that resulted in mtDNA copy number increase in *DGUOK* as well as *POLG* mutant cells at concentrations of 200 µM or higher without reporting any toxicity up to 800 µM. The potential benefit of nucleoside supplementation has not been investigated in *TWNK* mutant cells to our knowledge. Based on published data listed in Table 1, we supplemented both cycling (10% dFCS) and quiescent (0.1% dFCS) fibroblasts with individual (A, T, G, C) or combinations of deoxynucleosides (AT, GC, AG, TC, AC, GT, ATG, ATC, AGC, TGC, ATGC) at 200 µM concentration for 7 days. In line with previous investigators (Table 1), we used dialysed serum to maintain low exogenous nucleotide levels in the culture media. Previous authors suggested that supplementing Poly γ deficient cells with a combination of all four nucleotides is beneficial (Bulst et al., 2009). Given that our knowledge of nucleoside and nucleotide cycles and balances within cells is limited, especially in MDS (Ashley et al., 2007; Nikkanen et al., 2016) we wanted to explore the effects of more combinations of deoxynucleoside supplements. While the mtDNA content of POLG1, TWNK and even Control 1 fibroblasts doubled following supplementation with certain combinations (Figures 3A, B) we noted that simultaneously the cell numbers were lower for both Control 1 and POLG1 in 10% dFCS (see scattergrams in Supplementary Figure S3 showing that for 10% FCS cell numbers vs. mtDNA content the ungrouped *R* ^2^ ranges from 0.43 to 0.84). To visually represent the potential negative correlation between increased mtDNA content and low cell numbers we plotted the percentage change in mtDNA content and the percentage change in nuclear (cell) count on the same graph for each condition (Figures 3A, B). The treatments that simultaneously increased mtDNA content and reduced cell numbers included the combination of all four nucleosides favoured by previous investigators (Bulst et al., 2009). As well as significantly increasing mtDNA content across patients and controls (log transformed summed area of mtDNA nucleoids compared with baseline, all *p* < 0.001 with LMM, grouping by cell line), ATGC caused a ∼40% reduction in cell numbers in both control and patient cells and a concomitant reduction in mitochondrial membrane potential (MMP) (Figures 3C, D). This indicates that increased mtDNA content was associated with impaired mitochondrial function. In both POLG1 and TWNK fibroblasts the worst treatment combinations were ATG, TGC, GC, GT and G alone resulting in a reduced cell count by 50%–80% (Figures 3A, B). The treatment regime was similarly detrimental to healthy control cells. Supplementation of deoxy pyrimidines alone or in combination (C, CT or T) did not reduce mitochondrial membrane potential or cell numbers and were hence harmless to the cells. Of these, CT significantly increased mtDNA content in all patients and controls (log transformed summed area of mtDNA nucleoids compared with baseline, POLG2 *p* < 0.01, the rest all *p* < 0.001 with LMM), as did T or C alone in DGUOK cells (*p* < 0.01 with LMM). Supplementation of quiescent cells cultured in 0.1% dialysed serum with high doses (200 µM) of deoxynucleoside supplements also resulted in significant reduction in cell numbers (Supplementary Figure S4). However, the magnitude of the increase in mtDNA and of the reduction in cell counts was less than in cycling cells.

POLG2 cells in which Rho 0 cell counts reached 80% (Figure 1D) did not improve as much in response to any nucleoside combinations at 200 µM (not shown). As with POLG1 and control fibroblasts, supplementation with T, C and CT improved both cell counts and MMP in 10% FCS on POLG2 cells (not shown), but mtDNA content was only significantly increased in CT (*p* < 0.01 as above). CT also improved cell numbers but not MMP or mtDNA content in 0.1%FCS (not shown). The response of these cells to pyrimidine supplementation is consistent with their profoundly low respiratory chain activity and hence likely inability to synthesize them (Supplementary Figure S1; Figures 2A, C).

Given these results, we chose to proceed with a treatment regime using a lower deoxynucleoside concentration of 50 µM based on recently published promising results (Blazquez-Bermejo et al., 2019).

### Deoxynucleoside supplementation at 50 µM increases mtDNA content of quiescent patient cells without the damaging effects on cycling cells seen at 200 µM

**FIGURE 4:**
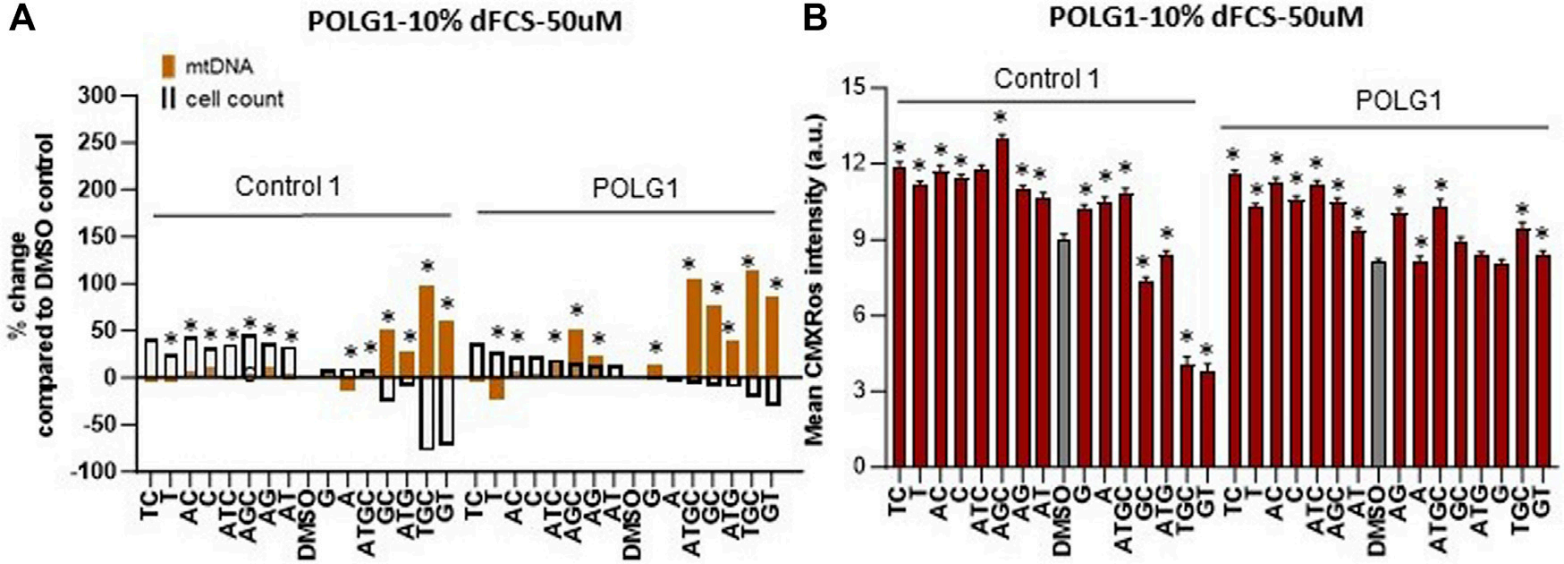
Deoxynucleoside supplementation of cycling cells (10% dFCS) at low concentration (50 µM). Effect of different nucleoside combinations on **(A)** mtDNA content and cell count and **(B)** CMXRos intensity. In **(A)**, effects are presented as average percentage change relative to DMSO baseline. Error bars are omitted because this ratiometric presentation of effects leads to large uncertainties. *, *p* < 0.05 compared to DMSO baseline using Mann-Whitney test. Supplementary Figure S3 shows patterns of statistical significance and number of cells in each sample. Further analysis with linear mixed models is used to assess statistical significance of group effects (see text). In **(B)**, significant values increase in mtDNA over baseline for illustrated run are indicated by * *p* < 0.05, (Mann-Whitney *U* test).

**FIGURE 5:**
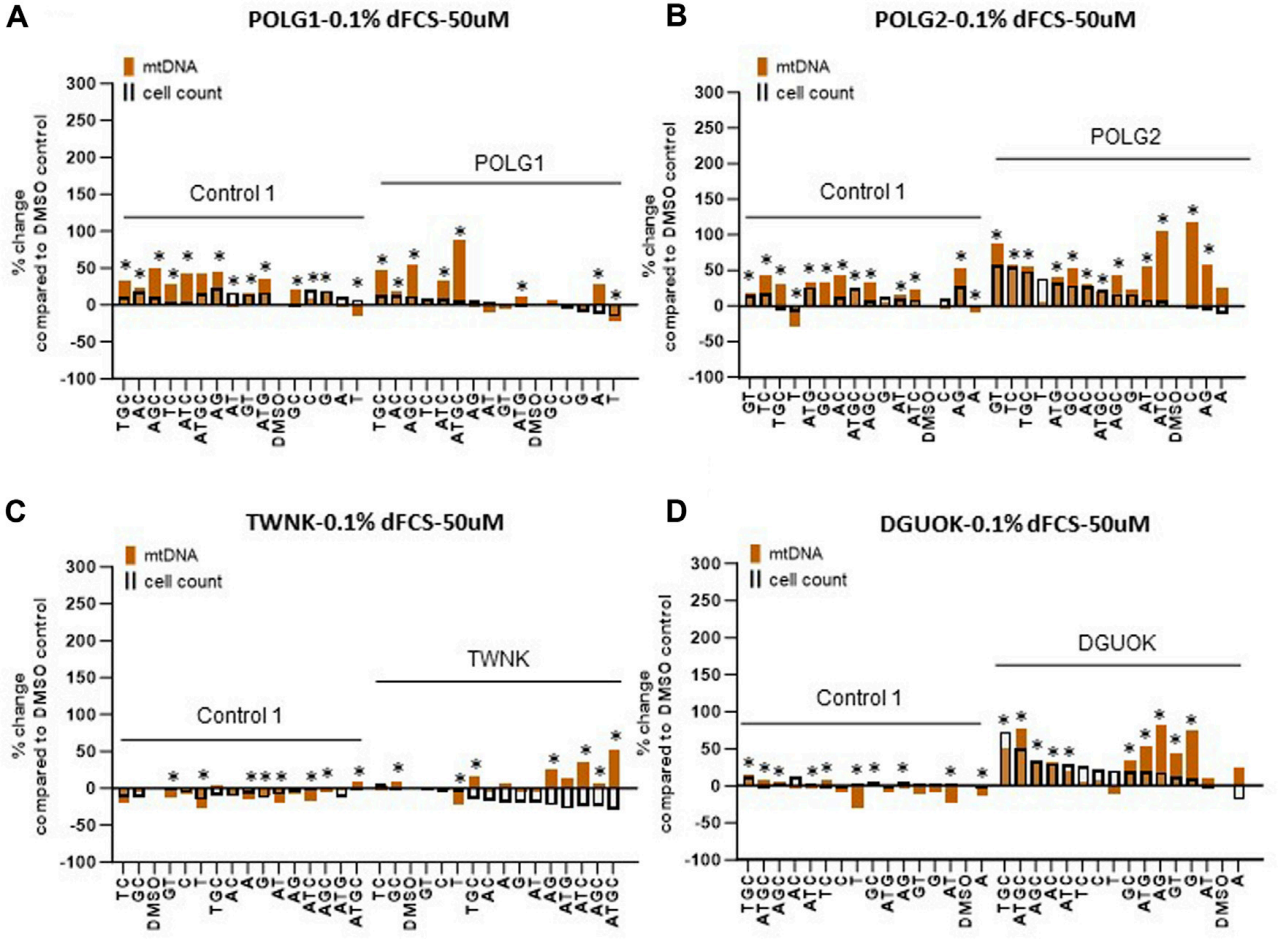
Deoxynucleoside supplementation of quiescent cells (0.1% dFCS) at low concentration (50 µM). Effect of different nucleoside combinations on mtDNA content and cell count in **(A)** POLG1 **(B)** TWNK **(C)** POLG2 **(D)** DGUOK. Effects are presented as average percentage change relative to DMSO baseline. Error bars are omitted because this ratiometric presentation of effects leads to large uncertainties. *, *p* < 0.05 compared to DMSO baseline using Mann-Whitney test. Supplementary Figure S3 shows patterns of statistical significance and number of cells in each sample. Further analysis with linear mixed models is used to assess statistical significance of group effects (see text).

Supplementation of cycling patient cells (10% dFCS) with 50 µM deoxynucleosides eliminated the toxic effects seen with 200 µM treatment (Figures 4A, B). To recapitulate the nucleotide levels of terminally differentiated cells (Bradshaw and Samuels, 2005) representing the most affected tissues in MDS, we followed the lead of previous authors by reducing the level of FCS in our medium to put the cells into a stationary growth phase. In replicating cells, most nucleotide components are synthesized by the *de novo* pathway, rather than the salvage pathway, used by stationary cells. Because the phase of the cell cycle substantially alters mtDNA content (Mandel et al., 2001), previous fibroblast models have used serum starvation (Magnusson et al., 2003). In the absence of serum, less than 1% of cells in a primary fibroblast culture pass through S phase (Taanman et al., 2003). Therefore, we proceeded to investigate the effect of nucleoside supplementation at 50 µM concentration in MDS fibroblasts cultured in 0.1% serum. MtDNA content in patient POLG1 cells was increased most consistently by the combination ATGC without a decrease in cell counts (Figure 5A). This treatment did not significantly improve cellular growth in POLG2 cells (Figure 5B). While all combinations of nucleoside supplements increased mtDNA content in POLG2 cells, due to their very low mtDNA content these percentage changes are misleading in this case. Therefore, we quantified the changes in the percentage of Rho cells in POLG2 cells following treatment (Supplementary Figure S5). Supplementation at 50 µM reduced the proportion of profoundly depleted Rho zeroes in POLG2 cells in both 10% and 0.1% FCS (Supplementary Figure S5). The mild reduction in Rho 0s with deoxypyrimidine supplementation in 0.1% FCS is again consistent with their profoundly low respiratory chain complexes and hence likely inability to synthesize them (Supplementary Figure S1). TWNK cells had increased mtDNA content in response to ATGC, however, cell numbers were reduced by ∼20% (Figure 5C). DGUOK fibroblasts responded well to most combinations of deoxynucleoside supplementation by increased mtDNA content, the most promising combination being ATGC (Figure 5D) in line with previous publications (Table 1). The number of cells analysed, and the *p*-value for the increase/decrease in mtDNA or mean CMXRos intensity over baseline in the experiments illustrated in Figures 3–5 is shown in Supplementary Figure S3.

### Analysis of cell morphology and relevance to reduced cell numbers

**FIGURE 6:**
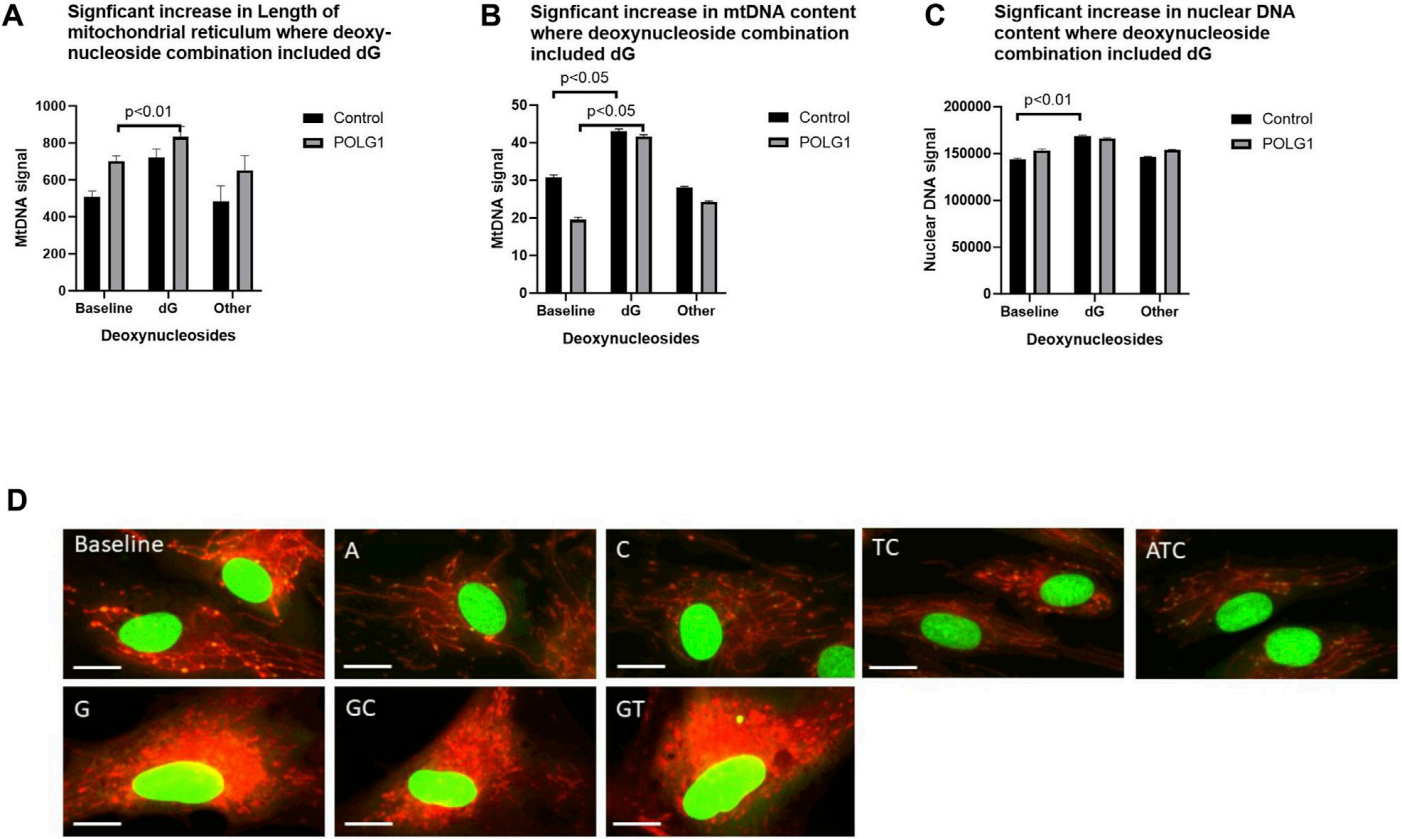
Effect of supplements that included 200uM deoxyguanosine on cell morphology. POLG1 and control cells were supplemented with 15 different nucleoside mixes (derived from data shown in Figure 2A). Supplements containing deoxyguanosine (G any) **(A)** increased the summed length of the mitochondrial reticulum, **(B)** cellular mtDNA content (integrated density, measured as sum of the grey levels of the PicoGreen signal) **(C)** and the nuclear DNA content (integrated density, sum of the grey levels of the PicoGreen signal) of both POLG1 and control cells compared to untreated cells. This was specific to supplementation containing G, as other mixtures did not. **(D)** Images of control fibroblasts stained with vital dyes PicoGreen and CMXRos showing abnormal mitochondrial reticulum morphology in cells treated with G-containing supplements. Error bars are SE. (Mann-Whitney *U* test). These data are also shown as Supplementary Figure S6E in which each individual condition appears as a separate point.

As well as finding that cell numbers were reduced in many of the treatments that included 200 µM deoxyguanosine (G any), we found that a substantial minority of G supplemented cells had increased area of their mitochondrial reticulum as well as increased mtDNA content, likely representing a proportion of cells in S phase (Figures 6A–D Supplementary Figure S6A). This was not, or was less apparent with 50 µM supplements (data not shown). We previously showed that the integrated density (the summed product of average signal intensity and nucleoid area) for PicoGreen signal is quantitative for mtDNA in wild type cycling cells (Ashley et al., 2005), and others that nuclear signal (Hoechst) correlates with phases of the cell cycle in high throughput imaging studies (Frolich et al., 2020). Hence, we validated our use of the integrated density of the PicoGreen nuclear signal as a semiquantitative measure of nuclear DNA using EdU (Roy et al., 2018) to pulse label S phase nuclei (Supplementary Figures S6B–C). The EdU labelling thus reflects our estimates of cell ploidy determined by PicoGreen labelling (Supplementary Figure S6D). Cells with nuclei with a high PicoGreen signal were apparent in baseline in 0.1% FCS in both patient and control (Figure 6A). This and the increased nuclear DNA content (Figure 6C) suggest that the proportion of these in S–G2 phase, rose significantly in POLG cultures supplemented with G containing combinations.

### Supplementation of quiescent cells with 50 µM ATGC following mtDNA depletion with ddC rescues mtDNA recovery

**FIGURE 7:**
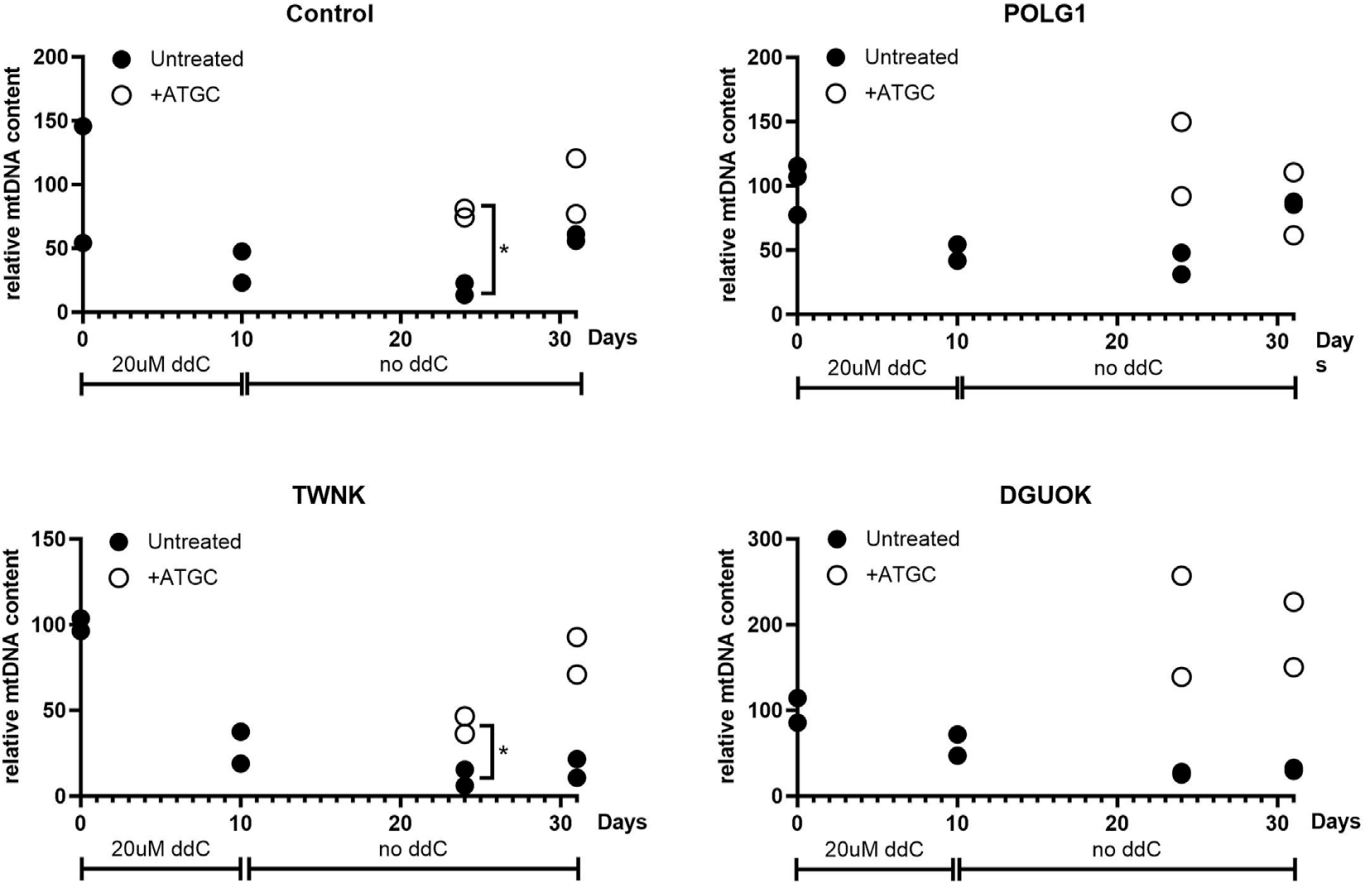
Quantitative PCR analysis of mtDNA content of quiescent cells subjected to mtDNA depletion and recovery with or without supplementation of ATGC at 50 uM. All fibroblasts were cultured in 0.1% dialysed FCS for 3 days followed by 20 uM ddC treatment for 10 days. Fibroblasts were left to recover for a total of 21 days in 0.1% dFCS culture medium with or without ATGC supplement. Supplementation with all four deoxynucleosides increased the rate of recovery in Control and POLG1 cells and rescued mtDNA recovery in TWNK and DGUOK cells. Each plot shows all of the data from two different experiments. Cells from patient TWNK and from the control that were supplemented with ATGC recovered significantly more rapidly than untreated, although the small sample size here means that this observation must be treated with caution (*p < 0.05, unpaired *t*-test). These data are also shown in Figure 8B, in a plot for comparison with the rate of labelling with heavy isotope.

Next, we investigated whether supplementation with ATGC could rescue mtDNA recovery following depletion, to further support our high-content imaging results. Cells were cultured in 0.1% dFCS high-glucose media for 3 days to induce quiescence, as previously (Taanman et al., 2003). This was followed by 10 days treatment with 20 µM ddC to deplete the cells of mtDNA. Following ddC treatment Control, POLG1, TWNK and DGUOK fibroblasts constantly cultured in 0.1% dFCS media were left to recover for 21 days either untreated or supplemented with 50 µM ATGC. Treated and untreated cell pellets were collected at the end of ddC treatment (d10) and at 14 (d24) and 21 (d31) days into recovery. Total cellular DNA was extracted from the samples and subjected to qPCR analysis to determine mtDNA copy number (normalised to baseline) at each time point (Figure 7). Following 10 days ddC treatment Control 3, POLG1, TWNK and DGUOK cells had and mtDNA content of ∼50% compared to untreated samples (Figure 7). The plots shown in Figure 7 are strongly suggestive of a treatment effect with ATGC. However, as we only have two replicates for each time course, individual t-tests between treatment and control only yielded *p* < 0.05 in two instances (control cells and TWNK, day 14). Statistical power here suffers from splitting the data by timepoint. In a preliminary attempt to take the data together, we used two-way ANOVA to block time effects and query the influence of treatment on mtDNA. Here, treatment effects were identified as significant (*p* < 0.01) in all cases except POLG (*p* = 0.40). It must be noted that the individual datapoints here are not fully independent and a more detailed statistical treatment would capture the time dependence more fully.

While mtDNA content reached pre-depletion levels by day 14 in the treated group, untreated cells were approaching similar mtDNA levels only at day 21. ATGC supplementation rescued mtDNA recovery in TWNK and DGUOK cells. MtDNA levels returned to 100% in TWNK cells and even exceeded 100% in DGUOK cells in the supplemented groups as opposed to the untreated cells which steadily remained at ∼50% without any sign of recovery.

**FIGURE 8:**
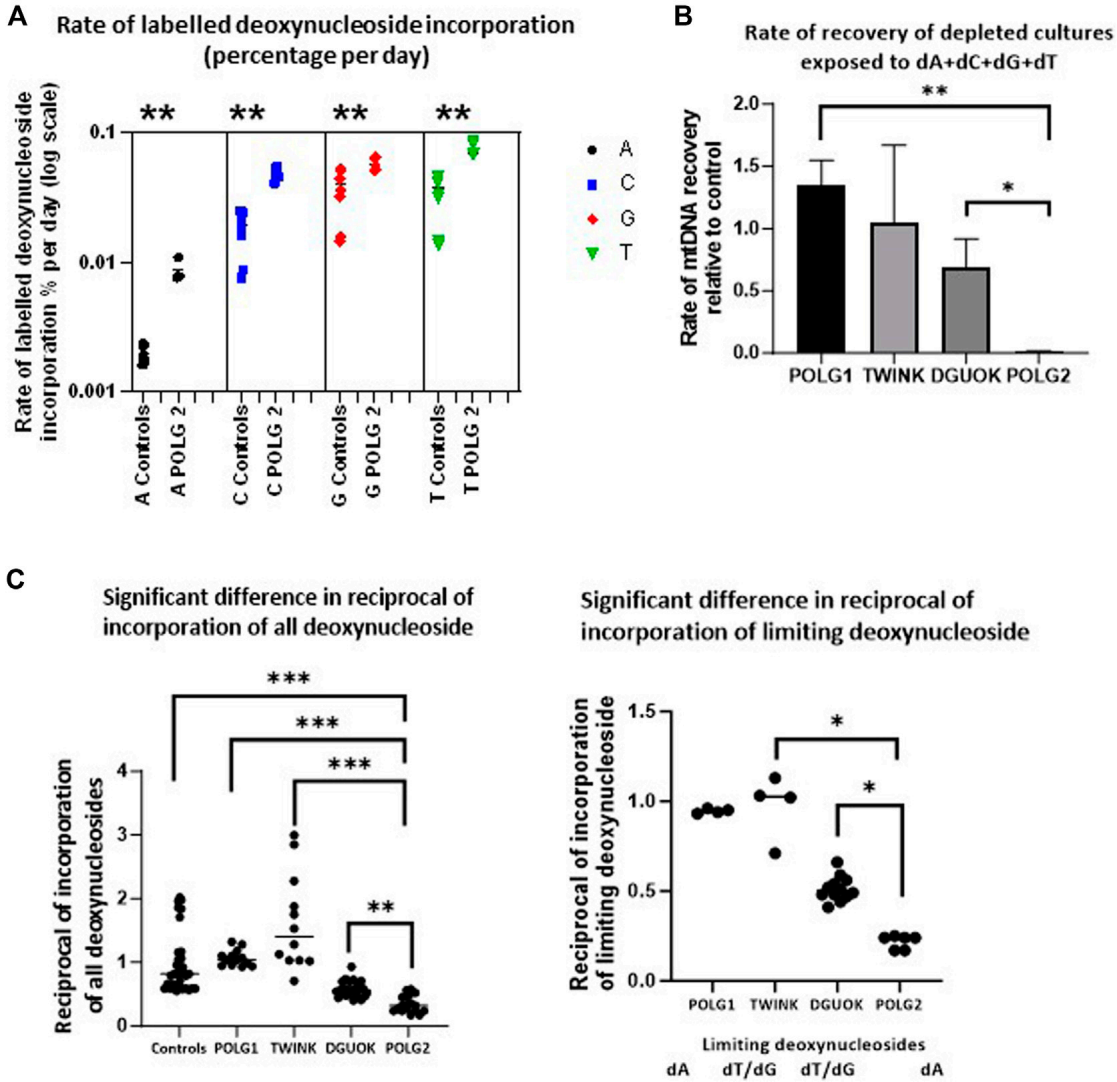
Incorporation of heavy labelled nucleoside supplements. **(A)** The rate of labelled nucleoside incorporation into fibroblast DNA of POLG2, patient with profound mtDNA depletion (baseline culture, not previously depleted with ddC). This suggests that the endogenous nucleoside pools are relatively depleted of A, C and T. ** *p* < 0.01, Mann-Whitney *U* test **(B)** Preliminary estimate of rate of recovery of mtDNA measured by qPCR derived from the experiment illustrated in Figure 7, following depletion with ddC for all except profoundly mtDNA-depleted culture POLG2, where this was baseline incorporation (error bars are SD), * *p* < 0.05, ** *p* < 0.01 *t*-test with unequal variances each experiment *n* = 2 **(C)** Median of suggested relative availability of limiting nucleosides for DNA replication using isotopically labelled nucleosides. The latter was estimated from the reciprocal of the rate of labelling of DNA with labelled nucleosides in the patient fibroblasts from each nucleoside supplement (left) and from the reciprocal of the rate of labelling of DNA in the patient fibroblasts normalised to the control median for the nucleoside that appeared to be limiting (right, marked below), plot derived from data shown in Supplementary Figure S7. The incorporation of isotopically labelled dNTPs as a proportion of cellular DNA reflects mitochondrial and cellular nucleoside pools. “dT/dG” indicates that there was no single limiting nucleotide because labelling was very similar for T and G. * indicates *p* < 0.05, ** indicates *p* < 0.01, *** indicates *p* < 0.001 (Mann-Whitney *U* test).

The depletion recovery experiment was repeated with each cell culture on 4 separate occasions. On two occasions the cells were supplemented with regular deoxyribonucleosides. These samples were used for subsequent quantification of mtDNA content. On two occasions the deoxynucleoside supplement mix consisted of 90% regular and 10% heavy labelled deoxynucleosides 13C A, 13C C, 15N13C T and 15N G. These samples were used to quantify the labelled exogenous nucleotides incorporated into DNA using Mass Spectroscopy (Figure 8A). We used the rate of incorporation of exogenous labelled nucleosides into DNA to probe the endogenous nucleoside pools under conditions favouring mtDNA synthesis. Figure 8A shows the rate of labelling of total DNA extracted from patient POLG2 fibroblasts (see Supplementary Figure S7 for the remaining three patients). We previously used short term pulse labelling of MDS fibroblasts with radiolabelled deoxynucleotides to show that the specific activity of newly synthesized mtDNA reflects the intra-mitochondrial nucleotide pool (Ashley et al., 2007). The ratio of labelled to unlabelled DNA in the whole cell DNA reflects the relative availability of the limiting nucleosides for DNA replication. These were most striking in the POLG2 cells derived from the POLG patient with the more severe mtDNA depletion, reflecting profound defect. The specific activity suggests that the dTTP pool was the lowest followed closely by dGTP, then dCTP and dATP was the highest in all the cultures (Supplementary Figure S7). Compared to the dNTP pools in the control (Figure 8B), the dATP pool was particularly low in POLG2, the severely affected patient, and the dCTP pool was also lower than the control. The milder patient POLG1 did not appear to have any deficiencies in the dNTP pool which appeared to be comparable to the control. Consistent with the expected impaired synthesis the dGTP specific activity of patient DGUOK was significantly higher than control (*p* = 0.014) but the dTTP incorporation suggested that the dTTP pool was also compromised (*p* = 0.005). As previously (Ashley et al., 2007), the reciprocal of the specific activity reflects the size of the pool of nucleosides available for DNA labelling. We therefore plotted the reciprocal of (i) the average specific activity normalised to control and (ii) the normalised specific activity that is highest in each patient culture (Figure 8C which should reflect the limiting nucleotide) and hence reflect the DNA synthesis rate (Figure 8B). This nicely validates the qPCR data showing rates of recovery from mtDNA depletion (Figures 7, 8B) suggesting that the labelled DNA reflects mtDNA synthesis and that the pools of available nucleosides is related to intramitochondrial availability. Because previous investigators found an excess of misincorporations at dTTP positions in the reference sequence of POLG iPS cells compared to controls (Hamalainen et al., 2019), we sequenced fibroblast mtDNA (average depth 5,000 reads). Sequence analysis of the POLG1 and POLG2 non-supplemented fibroblasts recovering from depletion by ddC did not show an excess over other misincorporations or over controls (not shown).

## Discussion

Mitochondrial DNA depletion syndromes often mean a devastating diagnosis with poor prognosis for patients. Currently, there are no available curative treatments. Nucleoside therapy using thymidine and deoxycytidine is currently in clinical trial for *TK2* myopathy (https://classic.clinicaltrials.gov/ct2/show/NCT03639701) and has been administered in patients under a compassionate use program resulting in improved clinical outcomes (Dominguez-Gonzalez et al., 2019). But the response to supplementation is determined by the precise molecular lesion. For instance, previous *in vitro* studies using *DGUOK* and *POLG* deficient cells reported partial restoration of mtDNA levels following nucleotide supplementation (Table 1). Building on previous findings we wanted to further explore nucleoside supplementation in MDS using patient derived cells with mutations in *POLG*, *DGUOK* and *TWNK*. To our knowledge the benefits of nucleoside supplementation have never been investigated in *TWNK* deficiency.

### High throughput imaging can assess cellular growth. mtDNA content. mitochondrial morphology and function

We supplemented cycling and quiescent fibroblasts from patients with 15 combinations of deoxynucleosides (A, T, G, C, AT, AC, AG, CT, CG, GT, ATC, ATG, AGC, TGC, ATGC) at high (200 µM) and low (50 µM) concentrations for 7 days. By using high-content imaging to measure changes in mtDNA content upon treatment we were able to quantify additional parameters of cellular wellbeing such as cell numbers, mitochondrial membrane potential and morphology, all linked to phenotypic severity ((Ashley et al., 2008) and Figures 1, 3). This technique enabled us to document a significant reduction in cell numbers (up to <90%) coupled with reduced membrane potential following supplementation at high deoxynucleoside concentrations in cycling cells, especially in the combinations that were most effective at restoring mtDNA content at 200 µM concentration (Figure 3). The most detrimental combinations of nucleosides were those that included deoxyguanosine, the only exceptions being the combinations AG and AGC. Furthermore, a substantial reduction in cell numbers was also evident in quiescent non-replicating fibroblasts treated at 200 μM, although to a lesser extent (up to 50%), indicating a combination of cytotoxicity and a prolongation in cell doubling time. This is entirely consistent with propensity of abnormally high dNTP levels to impair the fidelity of nuclear replication (Pai and Kearsey, 2017) and likely to cause cell-cycle arrest. The toxicity of supplemented deoxyguanosine was previously documented in Jurkat cells causing apoptosis via increasing dGTP as well as necrosis by depleting ATP (Batiuk et al., 2001). Furthermore, high-content imaging enabled us to investigate the effects of supplements at the level of single cells. We were thus able to group cells by nuclear DNA content and infer that the significant (*p* < 0.001) increase in nuclear DNA content in the cells treated with high-concentrations of deoxyguanosine containing supplements (G any) compared with untreated cultures was consistent with slowing of S/G2 phase, but not in cells treated with the other combinations (Figure 6; Supplementary Figure S5).

### Ameliorating toxicity by reducing nucleoside concentration

Due to the toxic effects of nucleoside supplements at high concentration (200 µM) we proceeded to supplementing cycling and quiescent fibroblasts with the 15 different combinations of nucleosides, reducing the concentration to 50 µM. We show that at 50 µM neither cell counts nor membrane potential are affected negatively, except for TGC, GT, GC combinations (Figure 4), that were the most detrimental at high concentrations as well (Figure 3). Hence the reduced concentration alleviated the toxic effects. We also found that T and T in combination with C were beneficial to fibroblasts from patient POLG1, when grown in 10% FCS, and T alone to profoundly depleted cells POLG2 (Figures 2–4). Neither benefited TWNK or control cells. The combination of deoxypyrimidines (CT) is used in commercially available MT1621 (Zogenix) (Amtmann et al., 2023) which was granted FDA Breakthrough Therapy and PRIME designations in 2018, for use in thymidine kinase deficiency. Our results suggest that this combination could be explored further for the treatment of POLG patients. In quiescent fibroblasts, a combination of all four nucleosides (ATGC) resulted in the highest increase in mtDNA content in POLG1, TWNK and DGUOK cells (all *p* < 0.001, Figure 5). Additionally, ATGC supplementation following mtDNA depletion with ddC significantly improved the recovery of mtDNA in these patient cells (Figure 7).

### Likely effects on nucleotide pathways

Exogenous isotopically labelled nucleosides were incorporated in cellular DNA, providing strong evidence that nucleosides can be taken up by the cell. Because the cells with the most profound mtDNA depletion, that have the slowest doubling time, acquire the highest proportion of label (Figure 8C; Supplementary Figure S7), we also have supporting evidence that exogenous nucleosides can be incorporated into mtDNA, and hence improve mtDNA copy number (Figure 7). It is well known that nucleotide imbalance, either excess or deficiency of nucleotides, can stall nuclear DNA replication (Pai and Kearsey, 2017) and derange nuclear DNA repair, check points and chromosome segregation. Increased *de novo* synthesis provides the nucleotides for the rapid pool expansion required for DNA replication, whilst allosteric regulation of ribonucleotide reductase (RNR) ensures that the synthesised dNTP pools are balanced. Cytoplasmic deoxyguanosine pools cannot be phosphorylated and will be converted to guanine by purine nucleoside phosphorylase (PNP, Supplementary Figure S7). Guanine is a substrate for the salvage pathway enzyme hypoxanthine-guanine phosphoribosyl transferase (HPRT) and is converted to GMP by transfer of a phosphoribosyl moiety from phosphoribosyl pyrophosphate (PRPP). The increase in GMP pools will inhibit the first committed step of purine *de novo* synthesis catalysed by glutamine phosphoribosylpyrophosphate amidotransferase (amidoPRT) (Kelley et al., 1975). PRPP is a driver of *de novo* synthesis by relieving inhibition on amidoPRT and reduced PRPP pools will contribute to reduced flux through the synthetic pathway. An unexpected result of incubation with G may therefore be that cytoplasmic dGTP pools are restricted, leading to inhibition or pausing of DNA replication and cell division in cycling cells. A mechanism of cytoplasmic dGTP pool restriction following G supplementation is consistent with the finding that adding all four nucleosides to the growth medium rather, than rescuing cell division, also led to a significant decrease in cell numbers, albeit to a lesser extent than supplementation with G alone. By contrast, supplementation with the purine deoxynucleoside A alone would be expected to have less of an effect on *de novo* purine synthesis as unlike G, A can be phosphorylated to the nucleotide, and this is indeed the case. Deoxyadenosine (which escapes this pathway) is converted to hypoxanthine by the sequential action of adenosine deaminase and PNP. Hypoxanthine is salvaged by HPRT to form IMP, consuming PRPP in the process and impacting on *de novo* purine synthesis (Supplementary Figure S7). IMP is able to replenish both adenine and guanine ribonucleotide pools, which may be of benefit to dividing cells. However, altering the balance between ATP and dATP pools may inhibit RNR activity and the capacity to expand dNTP pools needed for DNA replication (Hofer et al., 2012). The addition of pyrimidine deoxynucleosides to G in cycling cells caused a marginal further reduction in cell numbers compared to G alone, suggesting that dysregulation of *de novo* purine synthesis is, however, not the only mechanism for the reduction in cell numbers in the cycling cell model. It therefore seems likely that nuclear replication and/or mitosis was stalled in the large G-any treated cells.

### MtDNA content *versus* cellular toxicity

MtDNA synthesis can be uncoupled from the cell cycle because it uses dNTPs from the salvage pathway outside of S phase. Hence, mtDNA content can continue to increase, even if nuclear replication is stalled. It is conceivable that other processes such as inhibited mitophagy could also contribute to the increase in mtDNA content. Despite increasing mtDNA copy number, the G-containing supplements generally decreased mitochondrial membrane potential, suggesting that the additional mtDNA may be ineffective or damaged. Hence, many of the combinations of supplements in the G-any group are probably toxic both to nuclear and mtDNA synthesis.

## Conclusion

Using high throughput imaging to measure mtDNA content, we have added to the currently available data on supplementing patient cultures with nucleosides by screening 15 treatment combinations. Moreover, we have considered additional parameters of cellular health such as cell numbers and mitochondrial membrane potential to support the benefits of nucleoside supplementation in MDS fibroblasts with *POLG, TWNK* and *DGUOK* mutations. We conclude that, by including additional readouts of functional measures alongside mtDNA content, our assay significantly advances previous studies. In line with previous studies, we show that nucleoside supplements could benefit patients with *POLG* and *DGUOK* mutations. Additionally, for the first time we show that supplementation of all four nucleosides (ATGC) increases mtDNA content and supports mtNDA recovery following depletion in *TWNK* deficient fibroblasts. While supplementing the dNTP pool is helpful in specific disorders, these novel therapies must be used with care, to avoid damaging combinations.

## Summary

Nucleoside therapy is a promising treatment. Patients with mitochondrial DNA depletion syndrome (MDS), due to thymidine kinase two mutations, are supplemented with deoxy-pyrimidines. Previous studies have shown that supplementing polymerase gamma and deoxyguanosine kinase deficient myotubes with deoxy-nucleoside monophosphates increased mitochondrial DNA (mtDNA) copy numbers *in vitro*. We aimed to study the effects of different combinations of nucleosides in MDS patients. Therefore, we used fibroblasts harbouring mutations in *POLG*, *DGUOK* or TWNK (genes encoding alpha subunit of mtDNA gamma polymerase, deoxyguanosine kinase and Twinkle MtDNA Helicase respectively) to model the effects of 15 combinations of nucleosides, making this the most comprehensive cell-based study currently. Using high-content imaging, we measured the effects of supplements at the single-cell level on mitochondrial membrane potential, cell numbers and mtDNA content. We show that high concentrations of nucleoside combinations (200 µM), that increase mtDNA content, impair cell growth. This was alleviated by lowering the concentration to 50 µM. Furthermore, a combination of all four deoxynucleosides (ATGC) increased mtDNA content of POLG, TWNK and DGUOK quiescent fibroblasts, and significantly increased mtDNA recovery following artificial depletion using ddC. Both POLG derived replicating cell cultures benefited (effects on mitochondrial growth, membrane potential and mtDNA content) from C and T supplements, suggesting that this combination could be explored further for the treatment of POLG patients.

## Data Availability

The original contributions presented in the study are included in the article/Supplementary Material, further inquiries can be directed to the corresponding author.
