# Supplementary material for: Nucleoside supplements as treatments for mitochondrial DNA depletion syndrome
Source: Front Cell Dev Biol. 2024 Apr 2;12:1260496. doi: 10.3389/fcell.2024.1260496 (PMC11043827; doi:10.3389/fcell.2024.1260496)
Supplement: Supplementary file 1 [file DataSheet1.docx]

**Supplementary table 1:** Transitions and retention times used for the characterization of nucleosides ​
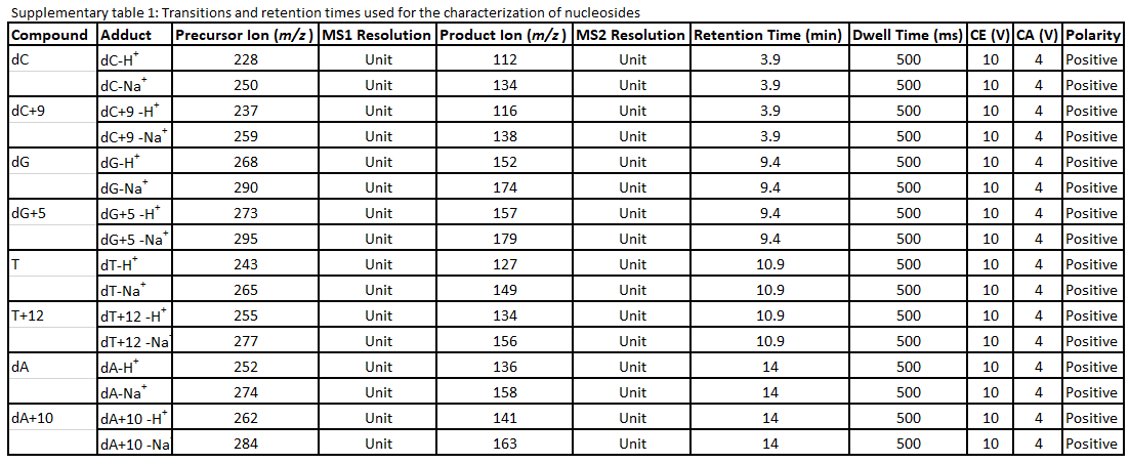


**Supplementary figure 1A**. Western blot analysis of fibroblast culture POLG2 and controls showing that protein expression of the electron transport chain subunits is reduced in POLG fibroblasts​.

MTCO1 is profoundly depleted in patient POLG2, the reduced abundance of subunits encoded by nuclear DNA is variable. n=2, representative image​. Total OXPHOS Human WB Antibody Cocktail (Abcam).


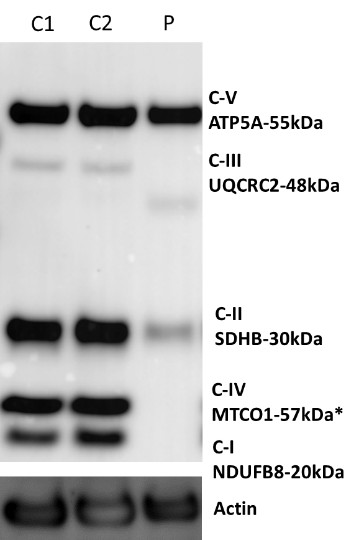


**Supplementary figure 1B.**

Images of fibroblasts from patient POLG2 showing that mtDNA-depleted cells are abundant compared with normal control. Arrows indicate profoundly depleted cells where mtDNA nucleoids were not or were barely visible. There is an unexplained systematic difference in the mean nuclear PicoGreen signal between patient and control culture. Size bar 37μM


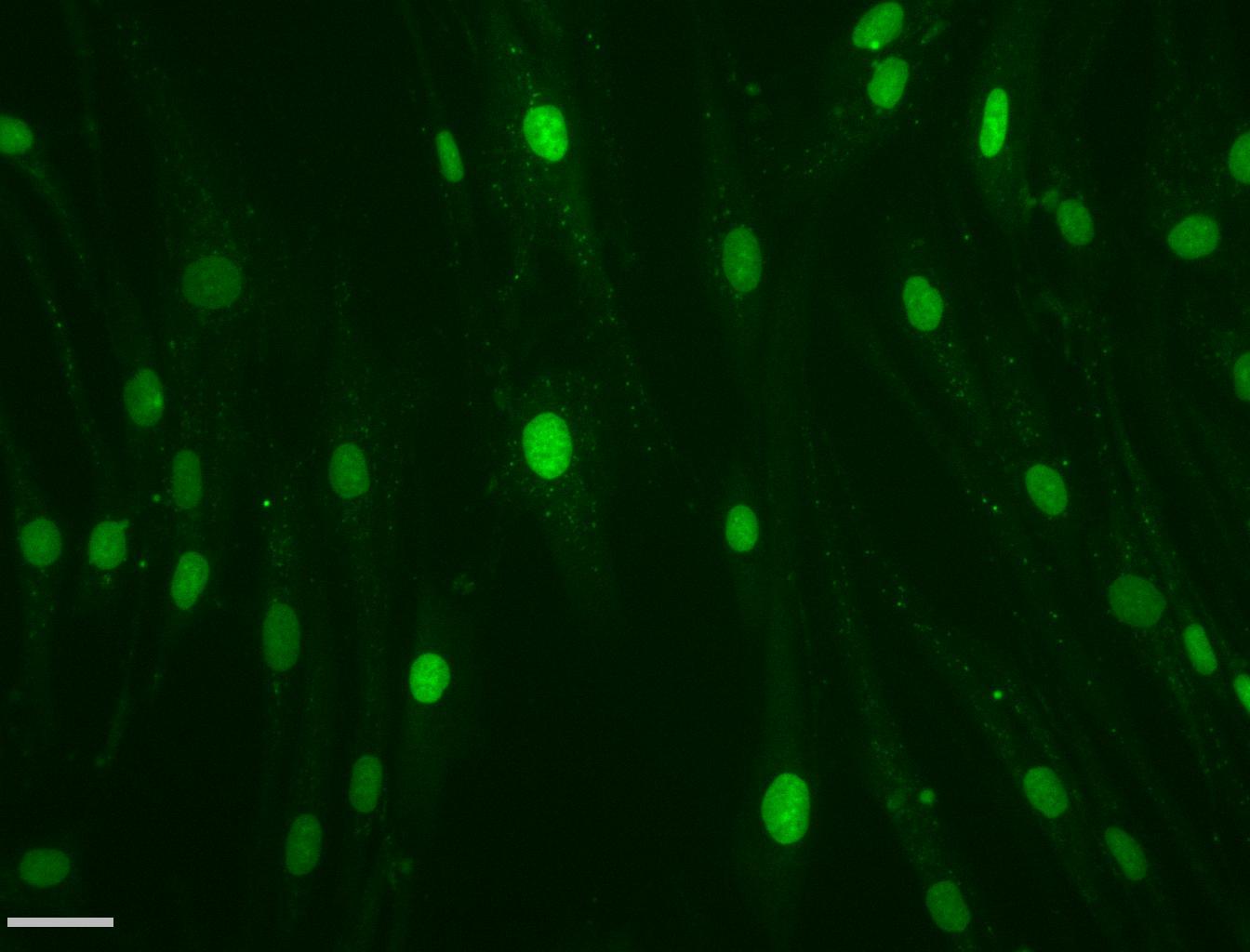

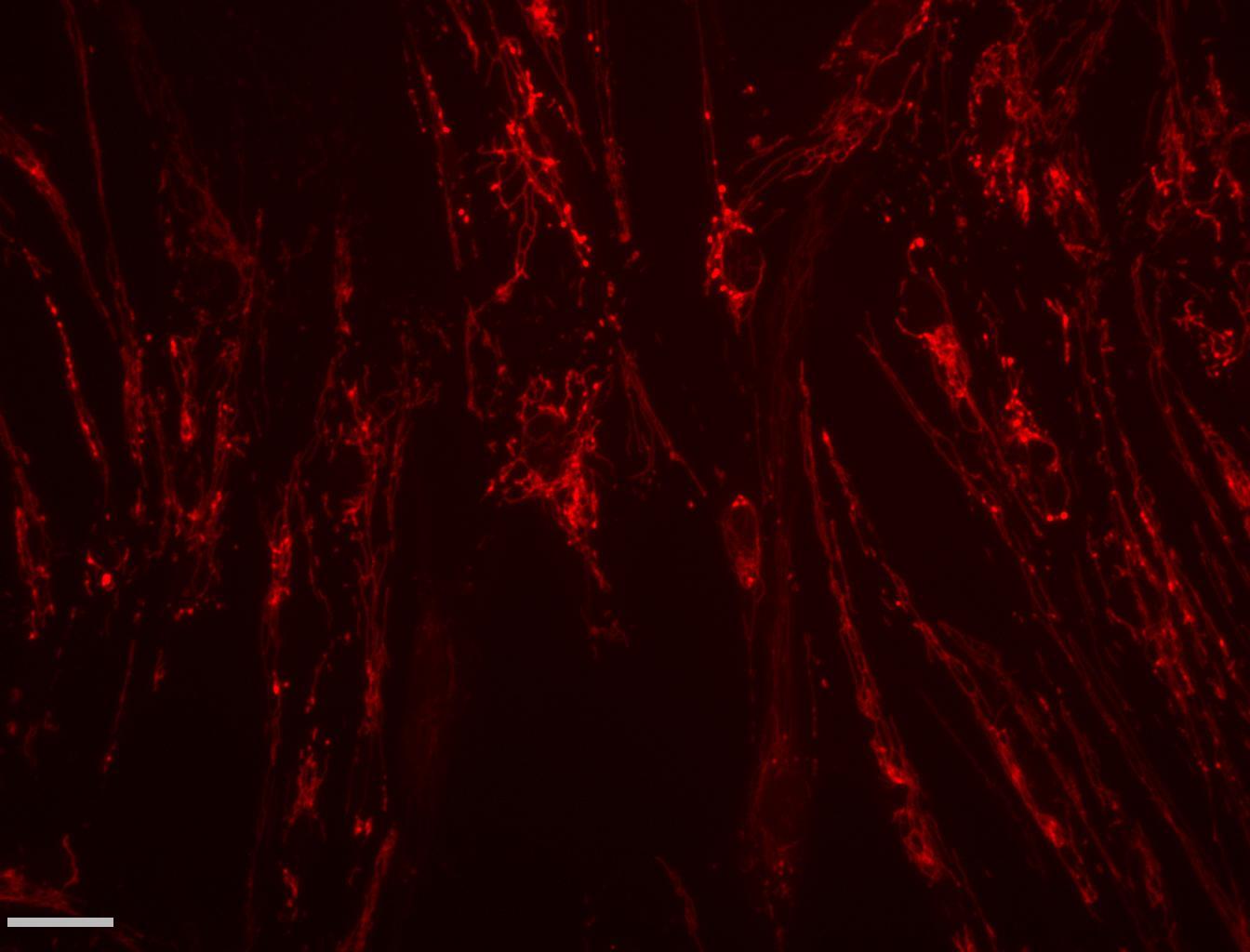

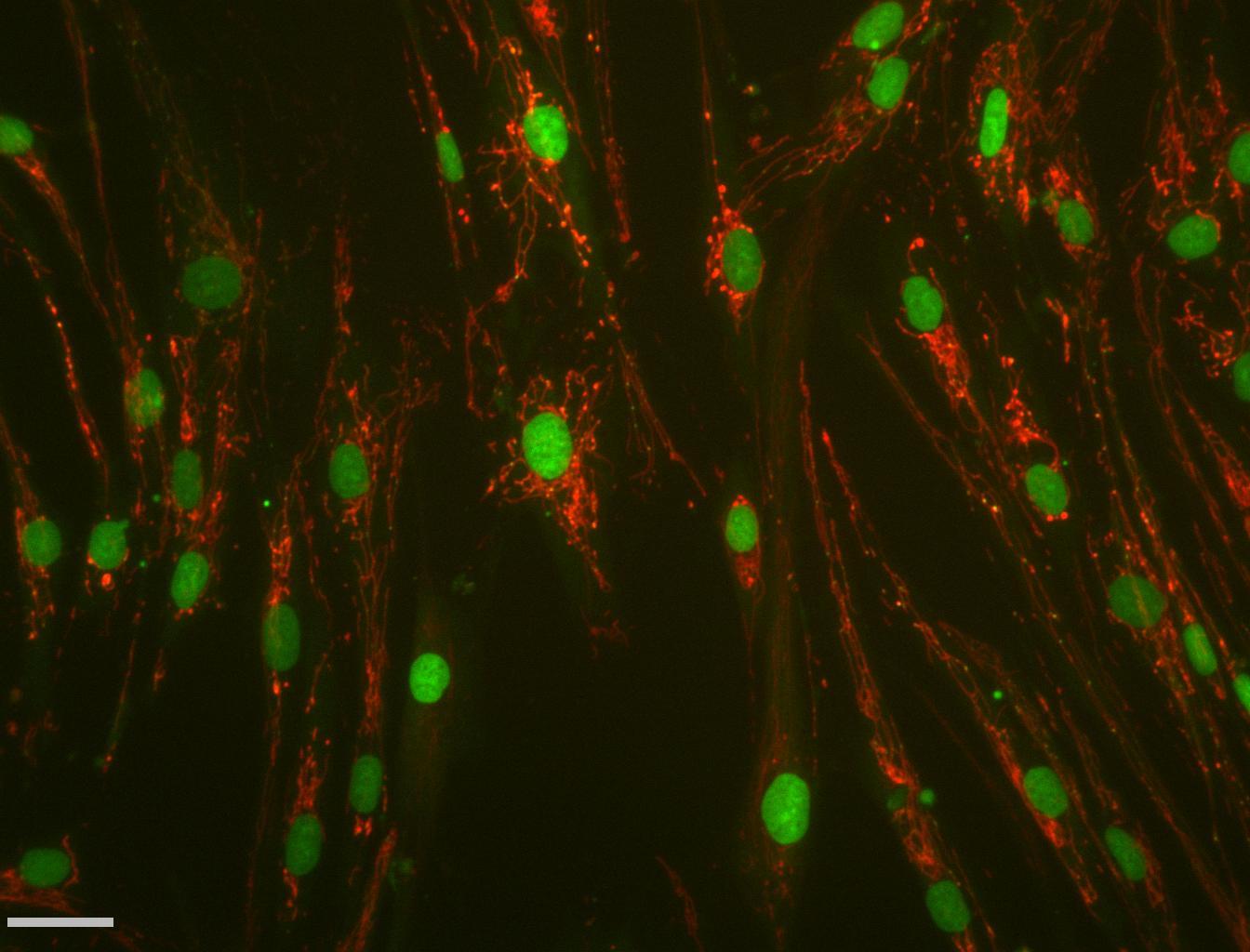

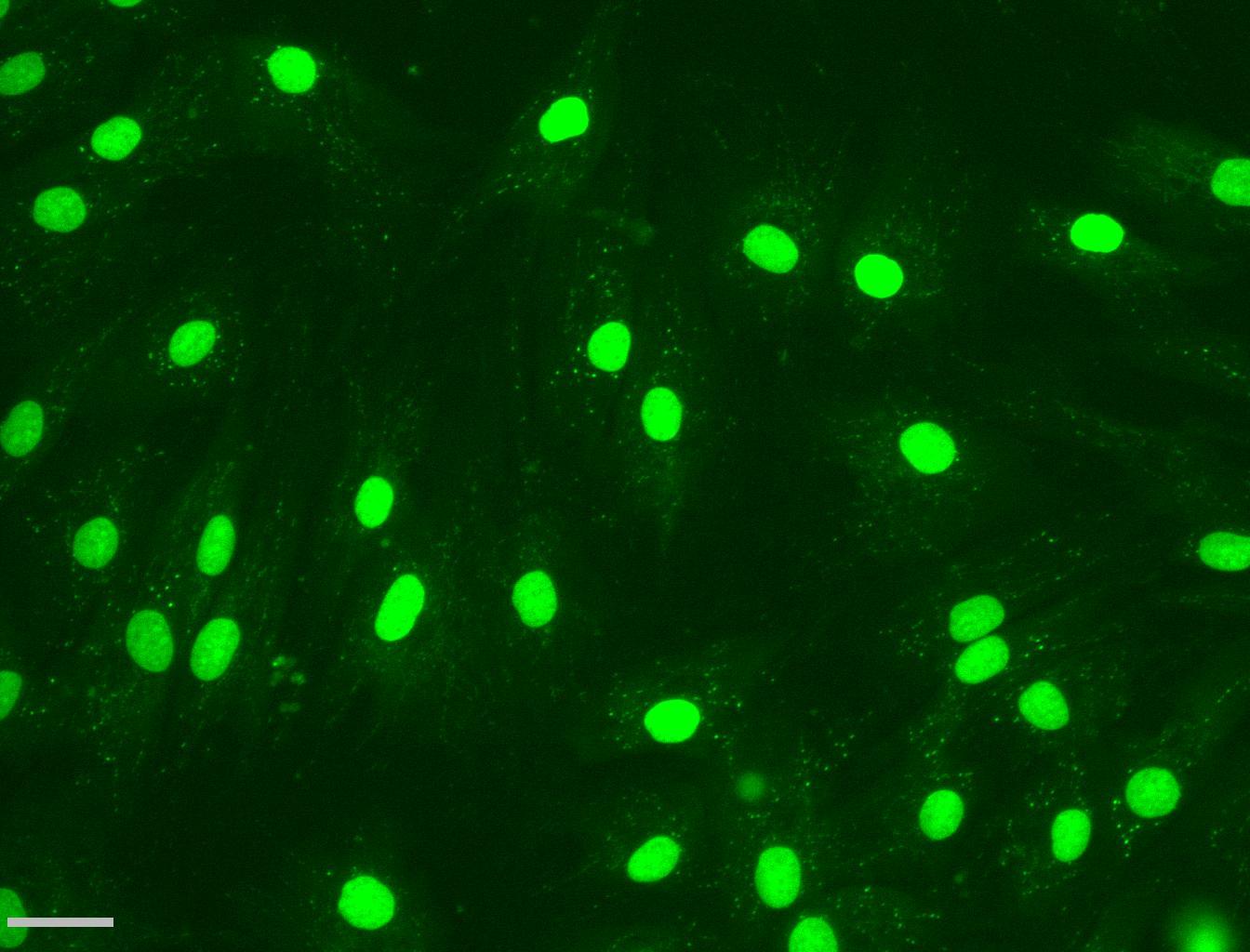

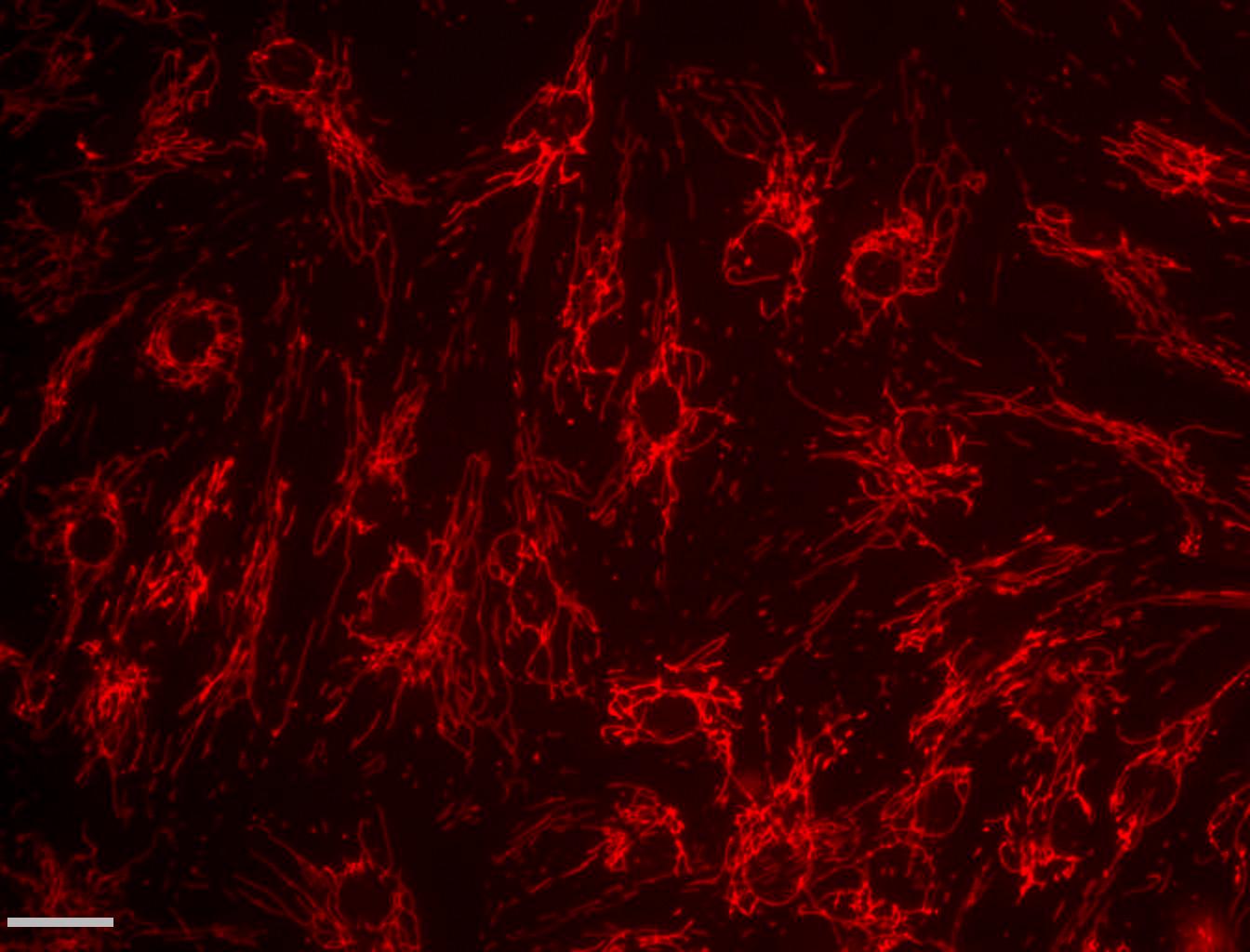

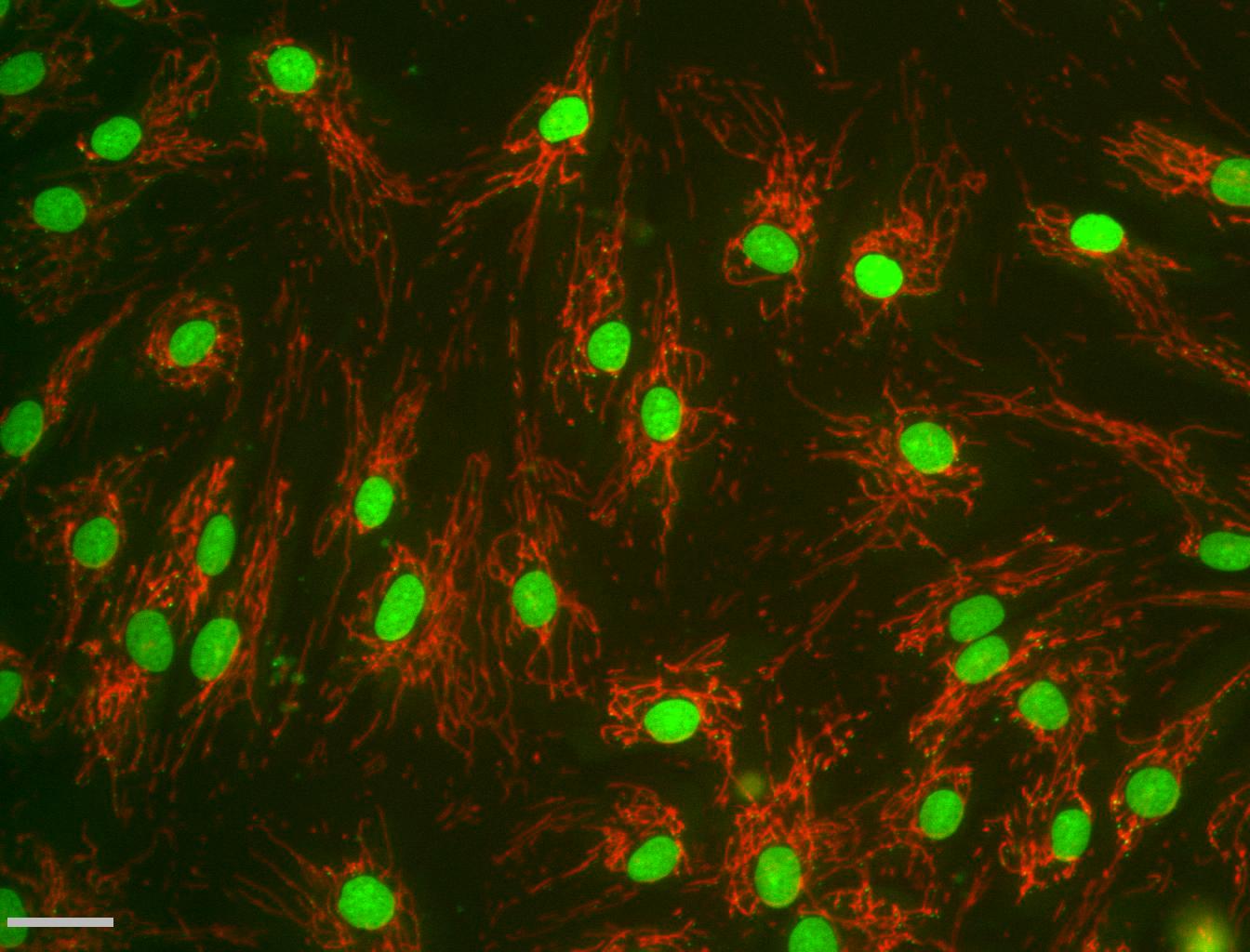


Control 2 POLG 2

Merge TMRM PicoGreen

**Supplementary figure 1C**

Scatterplot of PicoGreen staining (summed area of mtDNA nucleoids) against integrated density of TMRM signal (reflecting mitochondrial membrane potential (MMP) of patient POLG2 shows profound mtDNA depletion and depolarisation compared to control cells. There is a significant correlation between MMP and mtDNA content (Pearson correlation 0.7 p<0.001) Each point represents the mean of 200-300 cells.


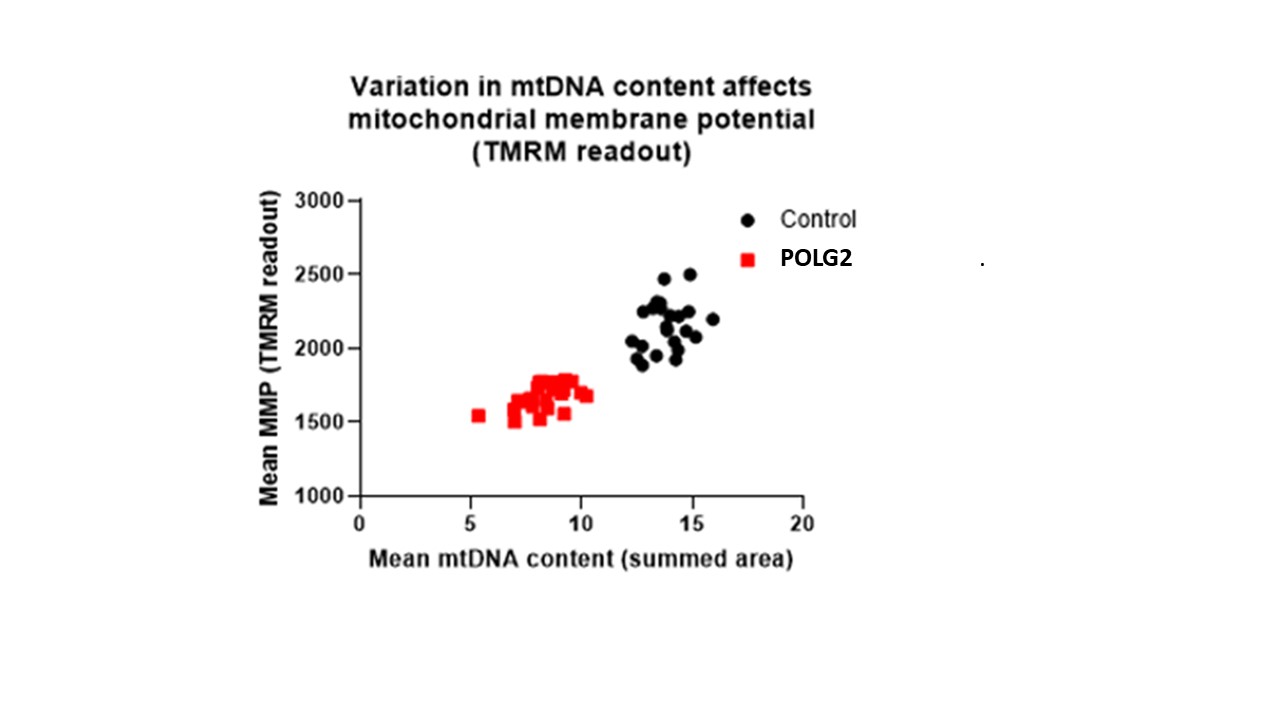


**Supplementary figure 2**. Validating the use of MitoTracker Red CMXRos instead of TMRM with PicoGreen for measuring mtDNA copy number, using high content imaging. CMXRos manifests less run-to-run variability than TMRM and CMXRos and TMRM can each be used with PicoGreen to measure mtDNA copy number. Measurements of mtDNA copy number (summed area of PicoGreen mtDNA signal) under different treatments within Control 1 culture counterstained with TMRM correlated well with PicoGreen signal counterstained with CMXRos. Bars represent 2 or 3 duplicates; error bars are standard errors.

**Supplementary figure 3**: additional information about Figures 3-5

To further support the visual impact of the data in figures 3-5, we have added scattergrams of mtDNA copy number against cell growth.


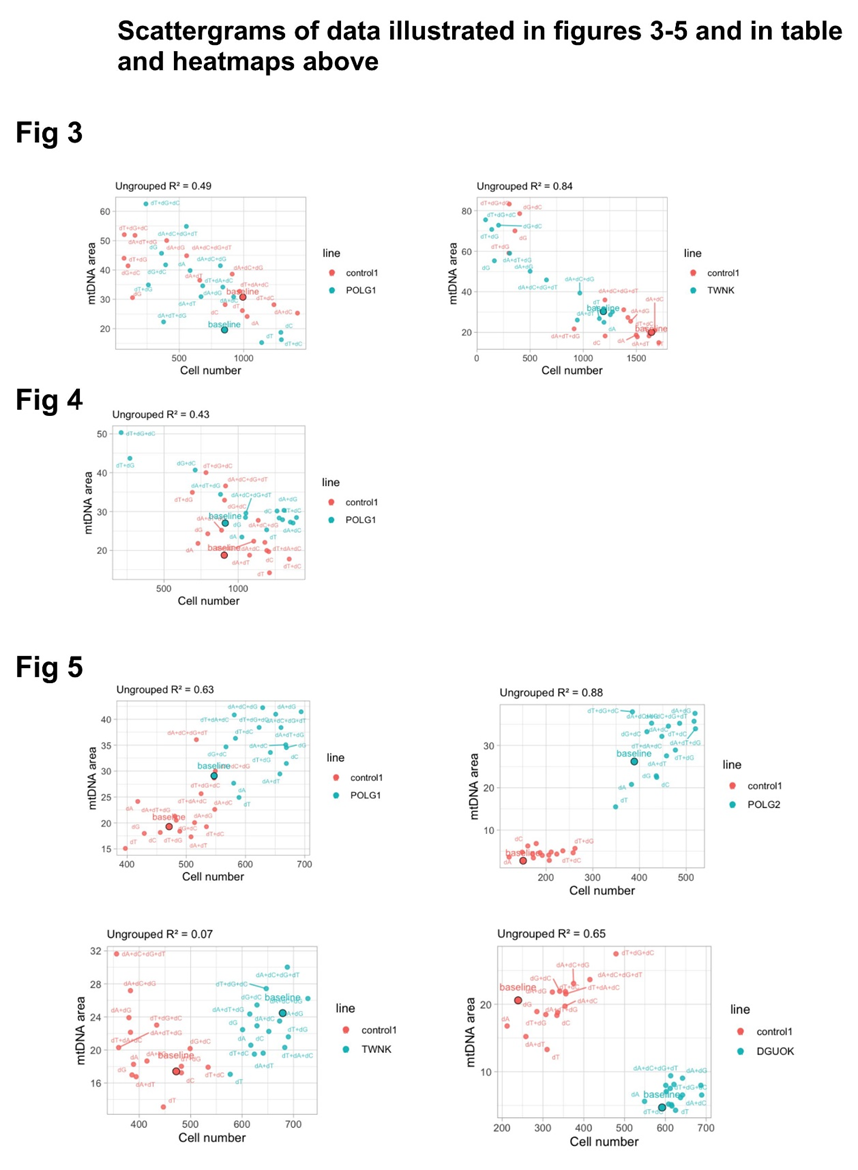


Table indicates cell numbers compared to baseline.

In the middle and bottom heatmaps, non-parametric testing was used to generate p values, because in most cases Shapiro-Wilk testing showed that the distribution differed from normality. Mann-Whitney U test (SPSS version 28) was used to test difference from baseline for technical replicates within a single run, and these values were converted into a colour scheme indicating increase/decrease over baseline. The middle table shows change from baseline summed area of mtDNA per cell and the bottom table shows change from baseline in CMXROS signal. “Increase” and “decrease” indicates p<0.001, “moderate increase” and “moderate decrease” indicates p<0.01 and “mild increase” and “mild decrease” indicates p<0.05.


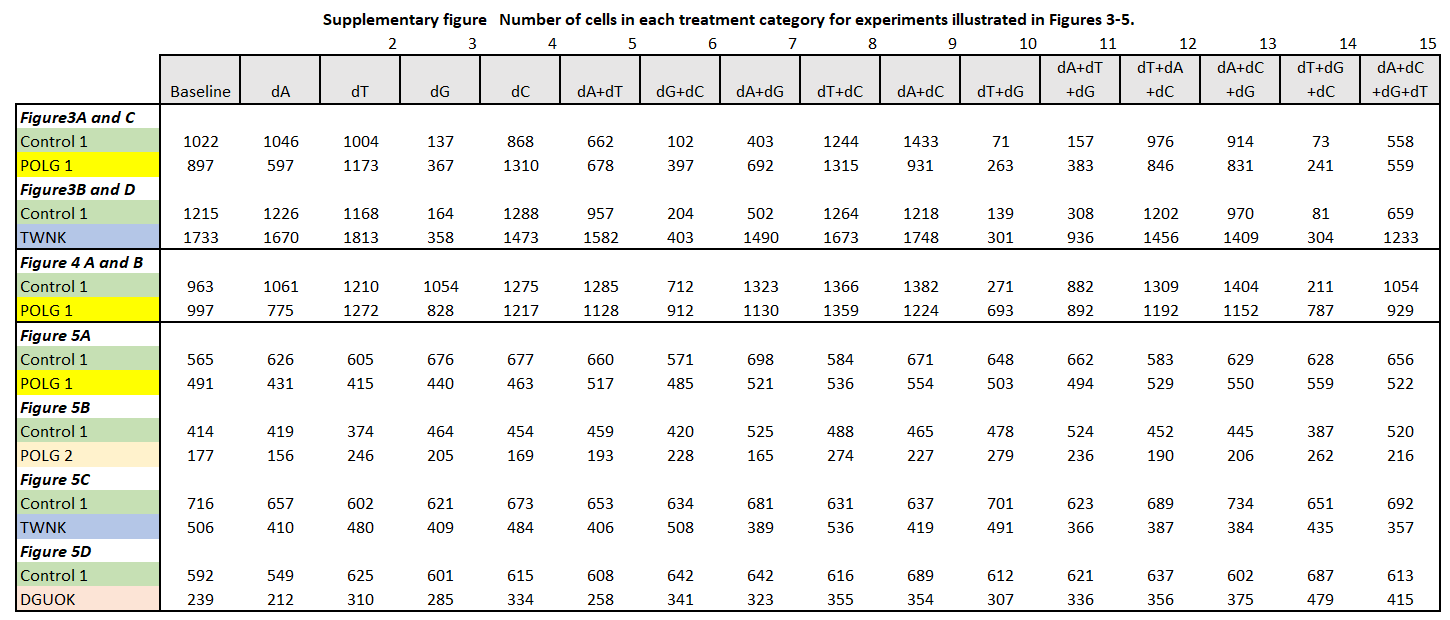


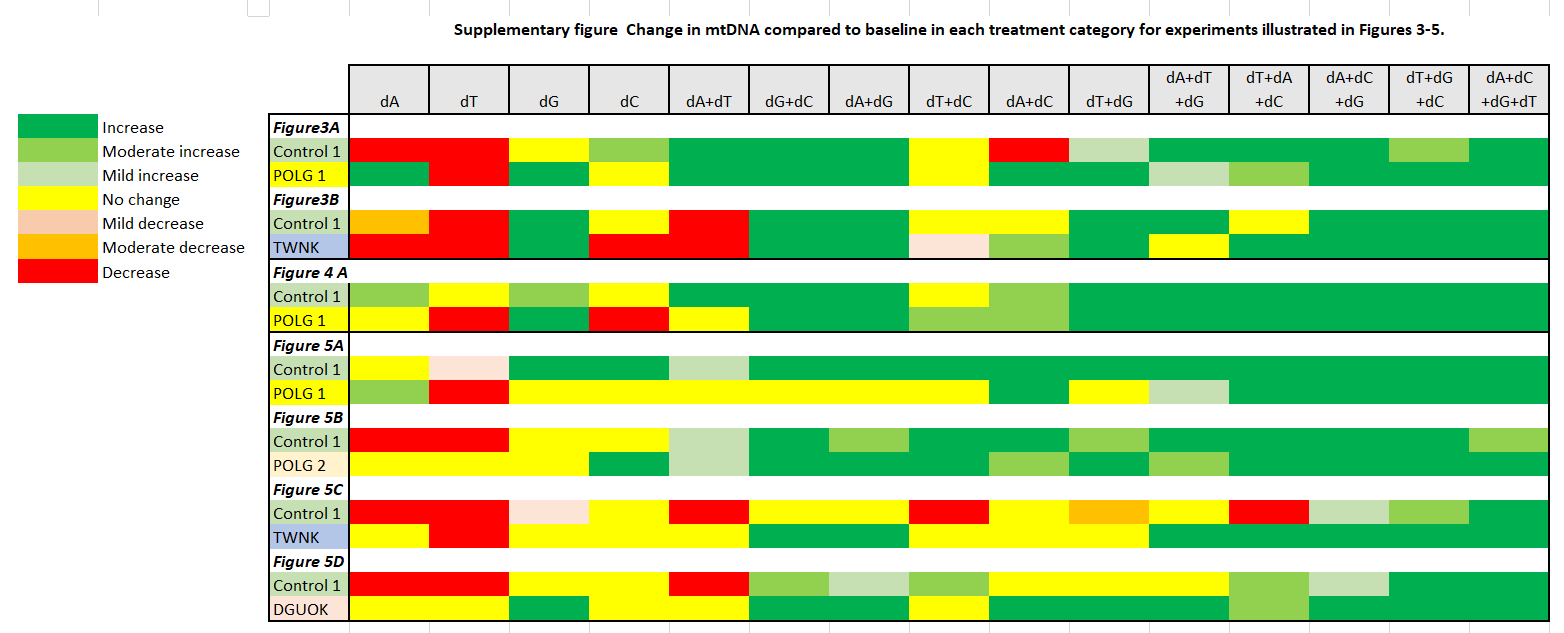


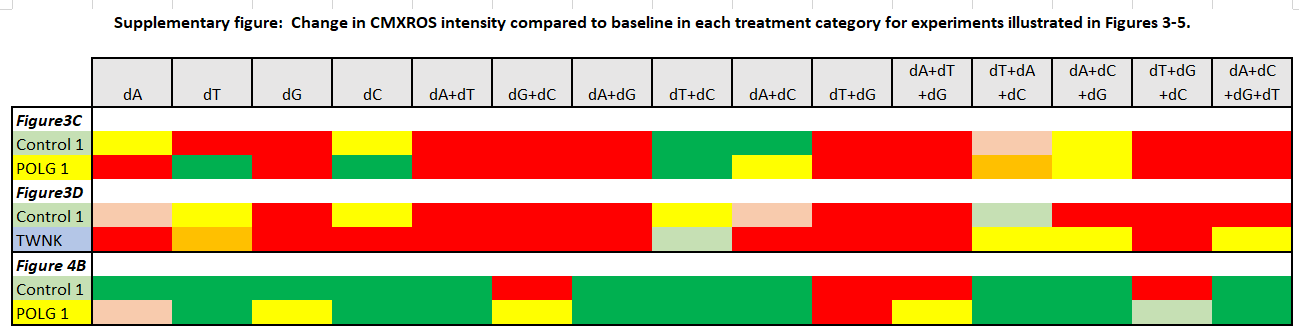


**Supplementary figure 4**. Deoxynucleoside supplementation of stationary cells (0.1% dFCS) at high-concentration of deoxynucleosides (200µM). Conditions are arranged by effect on patient cell count (pale grey bars) from best (left) to worst (right), percentage change in mtDNA content (brown bars) relative to baseline treatment.


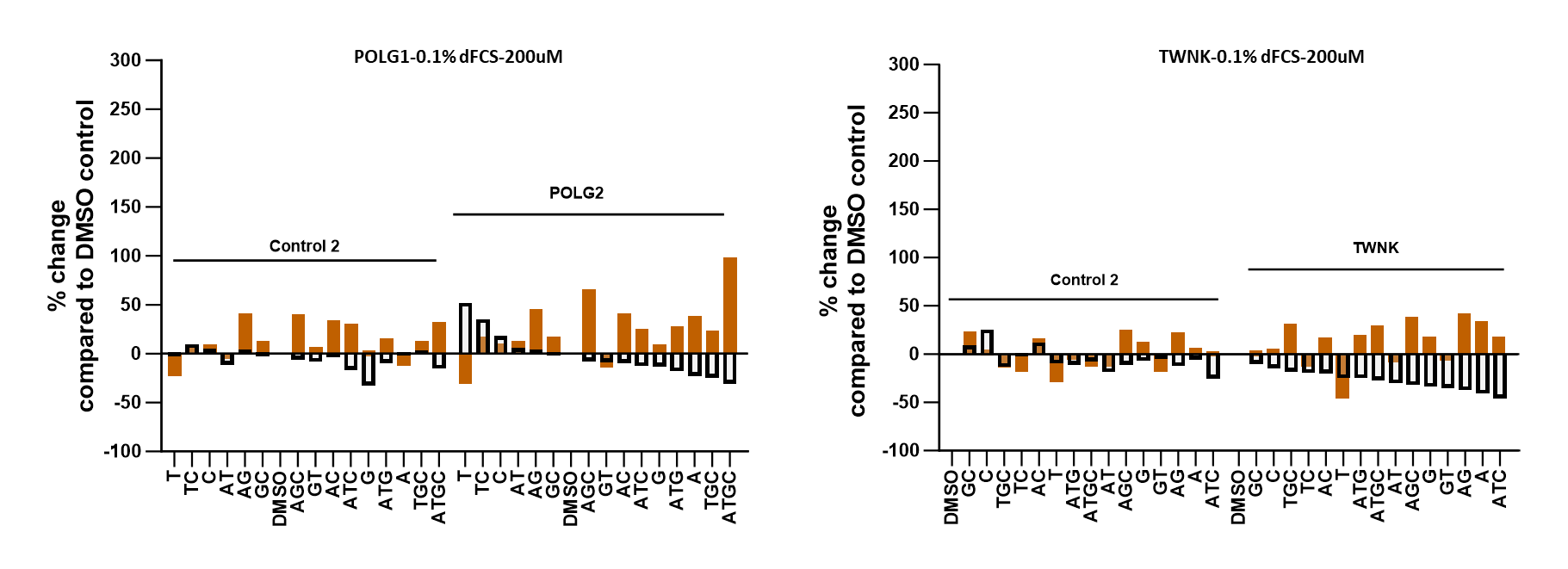


**Supplementary figure 5.** Percentage of Rho zero cells following deoxynucleoside supplementation in POLG2 cultures. In POLG2 cells the mtDNA content was profoundly low. Therefore, we plotted the percentage of Rho zero cells when cultured with 50um supplement mixtures in 10% dFCS and 0.1% dFCS.


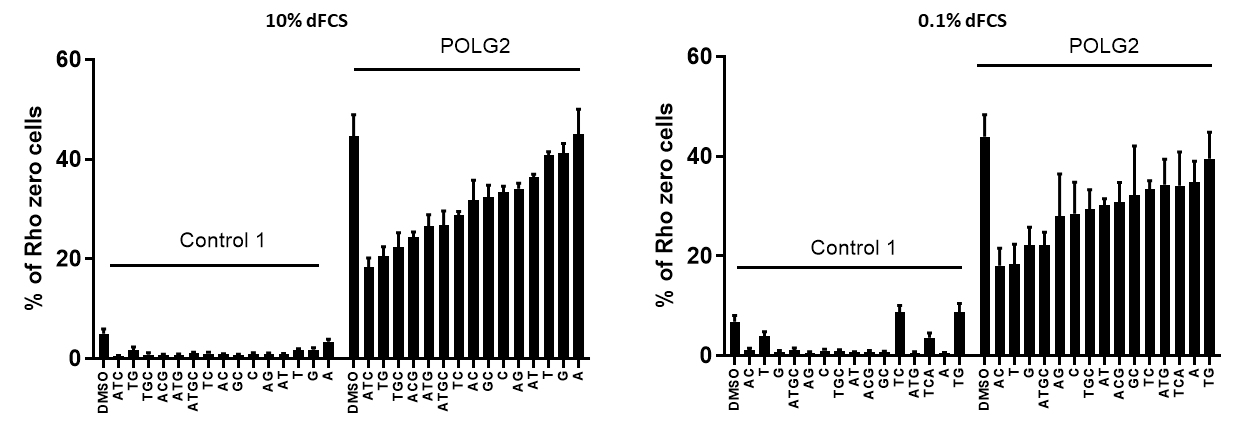


**Supplementary figure 6.** Validation of Nuclear Picogreen signal as an indication of nuclear DNA content

(A) Histogram of nuclear DNA content from PicoGreen signal from control fibroblasts in baseline conditions or with supplements at 200uM, either all conditions including dG or not including dG “Other”(0.1% FCS upper panel and 10% FCS lower panel). The distribution appears bimodal in the 0.1%FCS in the supplemented conditions. During routine use of serum deprivation to synchronise fibroblast cell cycle we previously observed a bimodal distribution in the integral of the nuclear PicoGreen signal (density x area) in the presence of 0.1% FCS that was not apparent in 10% FCS. This suggested that while the majority of cells had 2N nuclei, the minor peak harboured 4N nuclei. This distribution was apparent in both patient and control runs, whether or not cells had been supplemented.

(B) To explore whether the nuclei that appeared to have 4N DNA included S phase nuclei, we labelled control cells with EDU, an established protocol for detecting nuclear DNA synthesis (1). We used the modiﬁed thymidine analogue Edu Click-iT™ Alexa Fluor 594 kit (Invitrogen) to identify stages of the cell cycle. Cells were synchronised mechanically (2) and grown for 12, 20, 27, 36 or 42 hours and either EdU pulsed, fixed and stained with Hoescht and antibody to TOM20 (S1 and S2 correspond to cells with increasing EdU signal gating on EdU density x area and EdU area. Cells with an EdU signal with an area of <150 were deemed early S phase cells (henceforth S1) and >200 as late S or S2 cells.  Because the majority of S1 cells had DxA EdU<2000, cells with DxA EdU>4000 were also deemed to be S2 cells.

(C) Parallel cultures were live stained with PicoGreen (PG) and TMRM. Cells were imaged by INCell as above and parallel cultures compared. Using histograms of the density x area integral of the PG stained nucleus at various time points, 60,000 was selected as a cut off between 2N and 4N nuclei (ploidy). The bar chart shows that there were a higher proportion of 4N cells in early (left) than late (right) passage cells at corresponding time points, corresponding to their shorter doubling time.

(D) PicoGreen classification as 2N or 4N nuclei corresponds to expectation from the EdU labelling. Fibroblasts from control 1 and control 3 growing in 10%FCS (top) were gated on PicoGreen densityxarea as above for ploidy and (bottom) a parallel culture labelled with EdU . Area and density x area of the EdU signal (with an EdU signal with an area of <150) were deemed early S phase cells (henceforth S1) and >200 as late S or S2 cells as in B. Because the majority of S1 cells had DxA EdU<2000, cells with DxA EdU>4000 were also deemed to be S2 cells.

(E) The data in Figure 6 is replotted to show data points for individual conditions and the median value. POLG 1 and control cells were supplemented with 15 different nucleoside mixes (derived from data shown in Figure 3A). Supplements containing deoxyguanosine (G any) (i) increased the length of the mitochondrial reticulum, (ii) cellular mtDNA content (iii) and the nuclear DNA content of both POLG 1 and control cells compared to untreated cells. This was specific to supplementation containing G, as other mixtures did not. * p=0.02, **p=0.01 Mann-Whitney U test


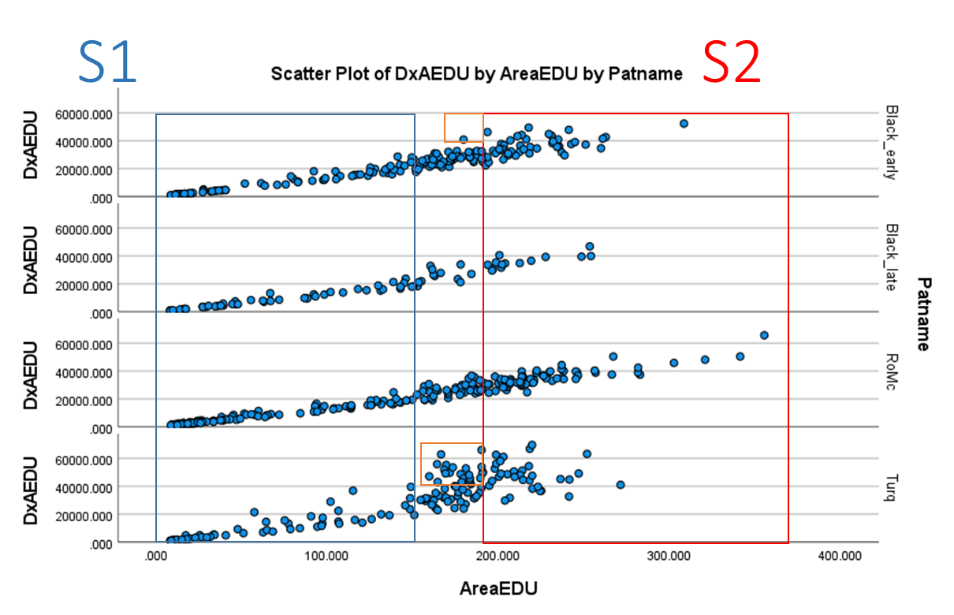

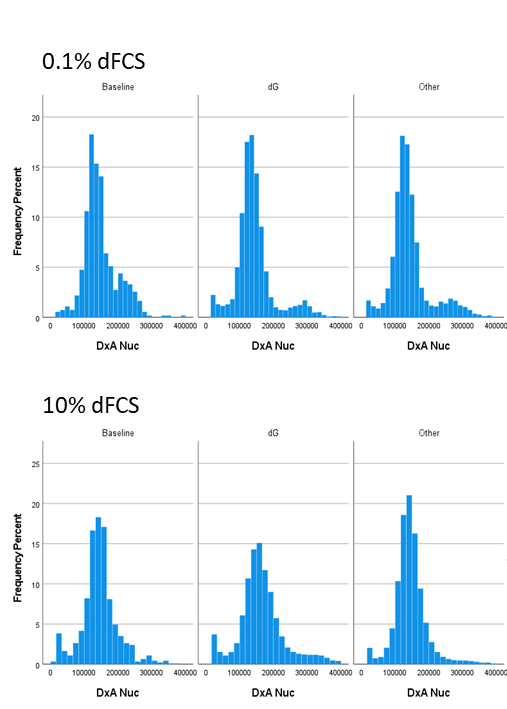
A B


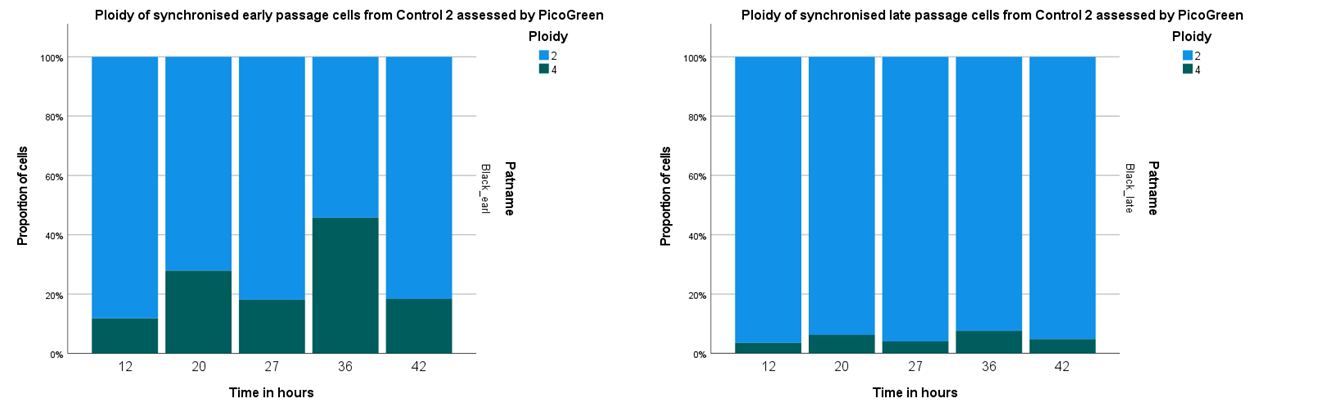
C


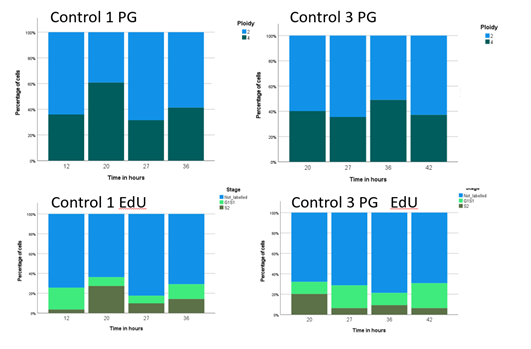
D

E(i) (ii) (iii)

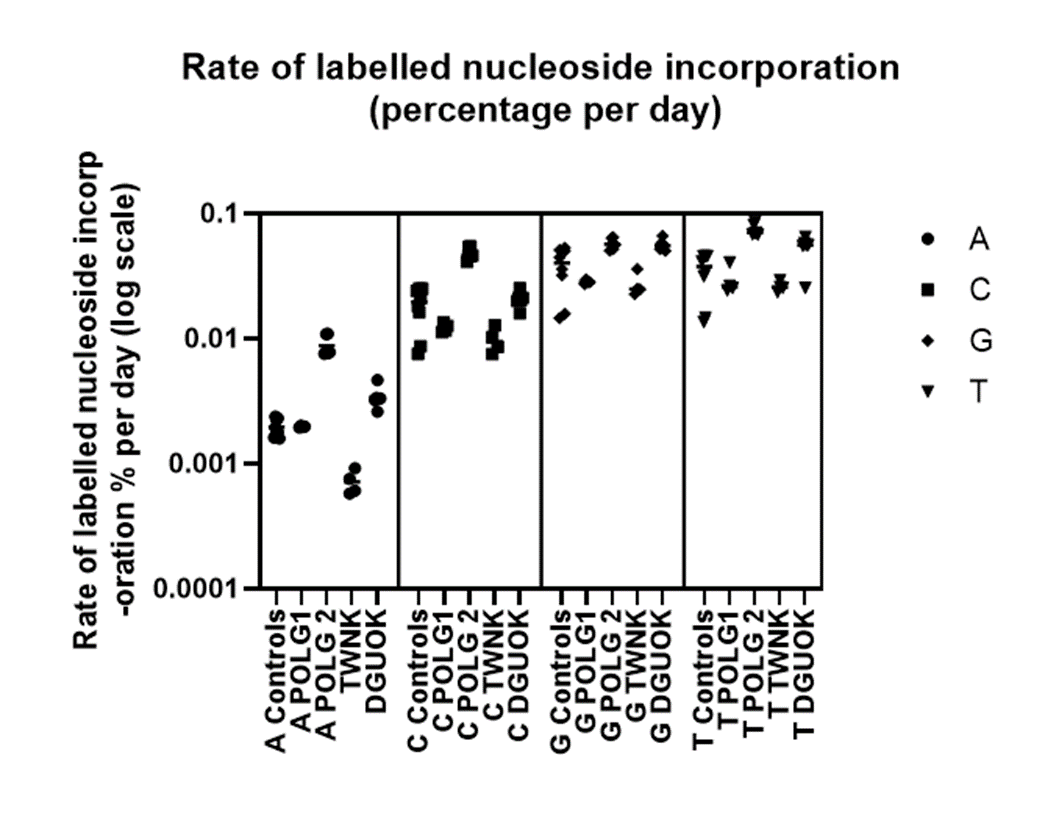
Supplementary figure 7. The rate of labelled nucleoside incorporation into fibroblast DNA of all 4 patients and control (same experiment as figure 8A). For POLG2 cultures the endogenous nucleoside pools are relatively depleted of dA, dC and dT, the other cultures are closer to control.

Supplementary figure 8. Salvage of supplemented purine deoxynucleosides consumes PRPP, restricting de novo purine synthesis. Purine deoxynucleosides are salvaged as the bases hypoxanthine and guanine to form IMP and GMP respectively. The salvage reaction consumes PRPP thus restricting de novo purine synthesis. Furthermore, the IMP and GMP formed are inhibitors of PRPP synthetase, further inhibiting purine synthesis.


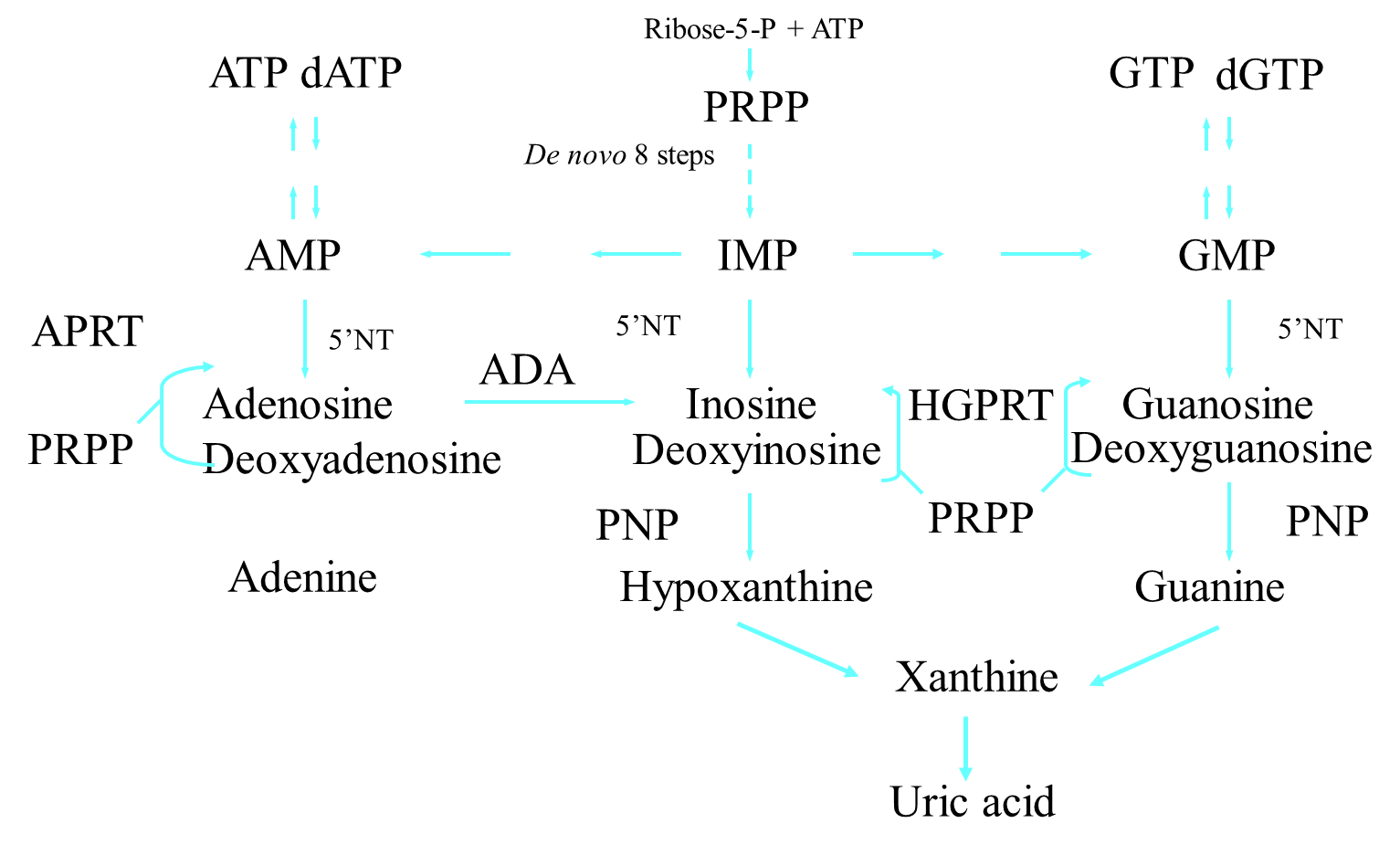
Key: PNP=purine nucleoside phosphorylase; PRPP=phosphoribosyl pyrophosphate; APRT =Adenosine phosphoribosyl transferase; HPRT (HGPRT)=hypo-xanthine-guanine phosphoribosyl transferase; ADA =Adenosine deaminase

**References for Supplementary information**

1. Roy S, Tomaszowski KH, Luzwick JW, Park S, Li J, Murphy M, et al. p53 orchestrates DNA replication restart homeostasis by suppressing mutagenic RAD52 and POLtheta pathways. Elife. 2018;7.

2. Terasima T, Tolmach LJ. Growth and nucleic acid synthesis in synchronously dividing populations of HeLa cells. Exp Cell Res. 1963;30:344-62.
